# Energy transfer within responsive pi-conjugated coassembled peptide-based nanostructures in aqueous environments†
†Electronic supplementary information (ESI) available: Characterization data for the peptides, Fig. S1–S7; and additional data (DLS data, energy-minimized models, TEM images, UV-vis/PL/IR spectra), Fig. S8–S28 (15 pages). See DOI: 10.1039/c4sc03122a


**DOI:** 10.1039/c4sc03122a

**Published:** 2014-12-05

**Authors:** Herdeline Ann M. Ardoña, John D. Tovar

**Affiliations:** a Department of Chemistry , Krieger School of Arts and Sciences , Johns Hopkins University , 3400 N. Charles St. , Baltimore , MD 21218 , USA; b Institute for NanoBioTechnology , Johns Hopkins University , 3400 N. Charles St. , Baltimore , MD 21218 , USA; c Department of Materials Science and Engineering , Whiting School of Engineering , Johns Hopkins University , 3400 N. Charles St. , Baltimore , MD 21218 , USA . Email: tovar@jhu.edu ; http://pages.jh.edu/chem/tovar ; Tel: +1 410 5166065

## Abstract

Steady-state and time-resolved photophysical measurements demonstrate energy transfer within π-conjugated peptide nanostructures composed of oligo-(*p*-phenylenevinylene)-based donor units and quaterthiophene-based acceptor units in completely aqueous environments. These peptide-based assemblies encourage energy migration along the stacking axis, thus resulting in the quenching of donor emission peaks along with the development of new spectral features reminiscent of acceptor emission. These spectral changes were observed even at minute amounts of the acceptor (starting at 1 mol%), suggesting that exciton migration is involved in energy transport and supporting a funnel-like energy transduction mechanism. The reversibility of nanostructure formation and the associated photophysical responses under different conditions (pH, temperature) were also studied. This unique material design incorporates two different semiconducting units coassembled within peptide nanostructures and offers a new platform for the engineering of energy migration through bioelectronic materials in aqueous environments.

## Abstract 2

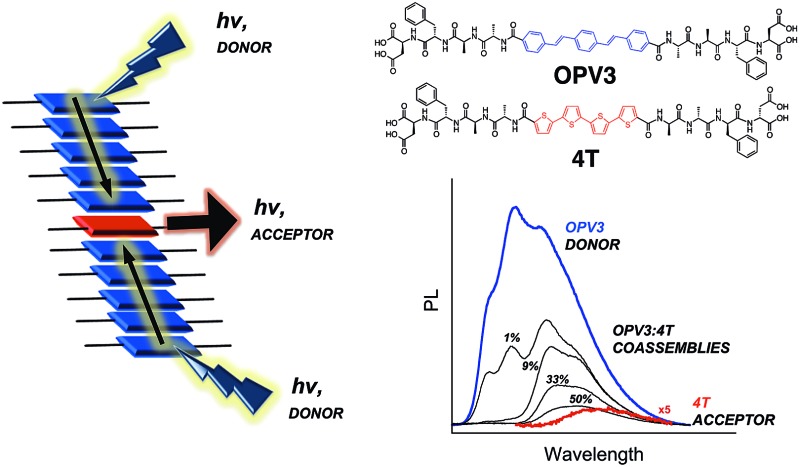
Energy transfer is demonstrated within a responsive donor–acceptor system which incorporates two different semiconducting units (oligo(*p*-phenylenevinylene and quaterthiophene) coassembled within peptide nanostructures in completely aqueous environments.

## Introduction

Supramolecular assembly is a recurring theme in nature that plays a significant role in the function of biological assemblies and is a widely used strategy to organize molecular components in organic electronic devices.^1^–^3^ In both photosynthetic complexes and in most optoelectronic devices, excitation energy transfer process plays a central role.^4^–^7^ This generally occurs *via* diffusion of excitons, which are electronically excited states of molecules that are created by light absorption or other electron transfer processes. These transport processes are highly dependent on ordering effects at different scales,^3^ whereby the direction and degree of intermolecular organization of π-conjugated assemblies are key factors that determine the efficiency of energy transport.^8^–^10^ The peptide-driven mesoscale organization of the chromophores in the photosynthetic membrane allows fast directional migration of excitation energy within the chromophore assemblies before being transferred to the reaction center (the final destination of the collected energy), which is crucial to the biological light-harvesting process.^11^–^14^ In order to maximize the harvesting capacity of pigments, an exciton funneling system is involved in the overall photosynthetic process, wherein different antenna complexes with distinctive transition energies are used to expand the spectral light absorption.^15^

It is therefore not surprising that multicomponent synthetic materials exhibiting supramolecular ordering and/or exciton migration, thus making good candidates for light-harvesting antenna mimics, have received considerable attention.^16^–^19^ Multichromophoric dendrimers^20^ and ring-like harvesting units^21^ are two among many molecular and other supramolecular forms that have been comprehensively studied in solution or in solid state (*i.e.*, as hydrogels or organogels)^14^,^22^ demonstrating the prospects for directional and near quantitative energy transfer in synthetic systems. Over the past decade, Meijer and coworkers reported exciton migration leading to energy transfer within supramolecular columnar stacks of oligo(*p*-phenylenevinylene) (OPV) in dodecane^5^ and hydrogen-bonded dimers between OPV–perylene bisimide in toluene.^23^ Functionalized OPVs that act as hydrogelators were also extensively studied for exciton funneling by Ajayaghosh and coworkers, showing that confining the acceptor units within an organogel made of molecular wires results in a more efficient energy transfer.^7^ Currently, studies involving molecular wires composed of π-stacked monomers or supramolecular nanostructured assemblies are of particular interest due to their generally superior carrier transport along the stacking axis, which is beneficial for nanoscale optoelectronic devices.^24^,^25^ Most studies of electronically responsive supramolecular analogs of biological structures are conducted in organic media; therefore, it is of great significance to explore aqueous supramolecular systems that could more efficiently mimic important bioenergetic processes under native conditions.^26^–^28^

Due to the intrinsic ability of peptides to self-assemble, several types of conjugated π-systems have been functionalized with peptides to further control specific intermolecular interactions that give rise to distinct assembly architectures and electronic delocalization.^29^–^35^ Previous studies on self-assembling peptides exhibiting energy transfer involve fluorescent dyes conjugated to peptide ends, showing efficient energy transfer in organic media or within hydrogels once a suitable acceptor dye has intercalated within the assembly network.^36^–^39^ Furthermore, the dyes often used in contemporary studies are chosen for their molecular photophysical properties rather than for their abilities to promote semiconductive electronic behavior.

In order to further demonstrate electronic function within peptide ensembles, we present here a two-component nanostructure composed of π-conjugated peptides containing donor and acceptor semiconducting π-units that upon self-assembly exhibit energy transport in completely aqueous media. This pH-responsive self-assembling peptide system also exhibits temperature-dependent aggregation behavior, which interestingly impacts the energy transfer process within the coassembled nanostructures. We present a nanomaterial design that affords the inclusion of two semiconducting units embedded within peptidic nanostructures and undergoes energy transport upon mixing and self-assembly under completely aqueous conditions, thereby providing a synthetic platform for energy harvesting and transporting aqueous nanomaterials.

## Experimental section

### General considerations

The chemicals used for 9-fluorenylmethoxycarbonyl (Fmoc)-based solid phase peptide synthesis (*N*-methylpyrrolidinone (NMP), *O*-(benzotriazol-1-yl)-*N*,*N*,*N*′,*N*′-tetramethyluronium hexafluorophosphate (HBTU), benzotriazol-1-yl-oxytripyrrolidinophosphonium hexafluorophosphate (PyBOP), *N*,*N*-diisopropylethylamine (DIPEA), Wang resin, and Fmoc-protected amino acids) were obtained from Oakwood Products, Inc. or Advanced ChemTech. Tetrahydrofuran (THF) was obtained from an Innovative Technologies PureSolv solvent purification system and stored over 4 Å molecular sieves (Sigma-Aldrich). *N*,*N*-dimethylformamide (DMF) was obtained from either Sigma-Aldrich or EMD Millipore Chemicals. DIPEA, THF and DMF were degassed by sparging with nitrogen (N_2_) gas for one hour prior to use. Tetrakis(triphenylphosphine)palladium (Pd(PPh_3_)_4_) was obtained from Strem Chemicals. The Biotech-grade cellulose ester dialysis tubings (MWCO 500–1000), with flat widths of either 16 mm or 31 mm, were obtained from Spectrum Labs. All other reagents and starting materials were obtained from Sigma-Aldrich and were used as received. 5-bromothiophene-2-carboxylic acid, 5,5′-bis-tributylstannyl-[2,2′]-bithiophene and 4,4′-((1*E*,1′*E*)-1,4-phenylenebis(ethene-2,1-diyl))dibenzoic acid were prepared using literature procedures.^40^–^42^ ^1^H NMR spectra were obtained using a Bruker Avance 400 MHz and the data was processed using Bruker Topsin 1.3. Chemical shifts are reported in parts per million relative to residual protio solvent [d_6_-DMSO *δ*: 2.50].

### General solid phase peptide synthesis (SPPS)

All peptides were synthesized using the standard Fmoc solid-phase technique with Wang resin pre-loaded with the terminal amino acid (Wang-Asp = 0.6 mmol g^–1^). To the resin in a peptide chamber, Fmoc-deprotection was accomplished by adding a (1 : 4) piperidine/DMF solution twice (successive 5- and 10-minute treatments) and then washing with NMP, methanol and dichloromethane (DCM). For the amino acid couplings, 3.0 eq. of the Fmoc-protected amino acid (1.0 eq. of the Fmoc-deprotected peptide bound to the resin) underwent external activation with 2.9 eq. of HBTU and 10 eq. DIPEA. The activated amino acid mixture was mixed for one minute prior to addition in the peptide chamber. The reaction mixture was allowed to mix for 60–120 minutes, after which was rinsed with NMP, methanol and DCM (3× each). The completion of all couplings was monitored using a Kaiser test on a few dry resin beads, repeating same amino acid coupling as needed. The general procedure for amino acid coupling was repeated until the desired peptide sequence was obtained.

### General *N*-acylation procedure for peptides

Following a procedure reported in the literature,^40^ a solution containing 2.1 eq. of 5-bromothiophene-2-carboxylic acid that was activated by HBTU (2.0 eq.) with DIPEA (10 eq.) was mixed for ∼3 h with the resin containing the completed peptide sequence. The resin was rinsed with NMP, methanol and DCM (3× each). The resin was treated again with 1.1 eq. of 5-bromothiophene-2-carboxylic acid that was activated by HBTU (1.0 eq.) with DIPEA (10 eq.) for 60 minutes. After rinsing the resin with the standard wash cycle (NMP–methanol–DCM, 3× each), completion was assessed using a Kaiser test on a few dry resin beads. Treatment with 1.1 eq. of the activated 5-bromothiophene-2-carboxylic acid was repeated as needed.

### General on-resin Stille coupling procedure

Following a procedure reported in the literature,^40^ the *N*-acylated peptide made by following the general procedures described above were transferred to a Schlenk flask equipped with a reflux condenser. The dried resin with Pd(PPh_3_)_4_ (4.0 mol% relative to the amino acid loading in the resin) was kept in the Schlenk flask under a nitrogen (N_2_) atmosphere (∼10–20 mTorr). In a separate vessel, a ∼15 mM solution of 5,5′-bis-tributylstannyl-[2,2′]-bithiophene was prepared in DMF. This was then added to the reaction flask *via* syringe. The reaction mixture was heated up to 80 °C while agitating by constantly bubbling nitrogen (N_2_) gas in the solution. The said conditions were maintained for ∼16 h, and then the reaction mixture was allowed to cool to room temperature. The resin was washed with DMF (3×) in a peptide chamber, followed by the standard wash cycle. The synthesized π-conjugated peptides were then subjected to cleavage procedure.

### General cleavage procedure for peptides

The cleavage cocktail was prepared with 9.5 mL of trifluoroacetic acid, 250 μL Milli-Q water, and 250 μL of triisopropylsilane. The resin was treated with 10 mL of cleavage cocktail in a peptide chamber for 3 hours. The filtrate was drained and the resin was washed with DCM (3×). The filtrate was concentrated under reduced pressure. The crude peptide was precipitated out of the filtrate by adding 90 mL of cold Et_2_O, allowing the suspension to sit for 5 minutes at 4 °C. The pellet formed was isolated by centrifugation, followed by decanting the solvent and drying the solid formed. The pellet was redissolved in Milli-Q water with a few drops of ammonium hydroxide (to completely dissolve the solid) and was subjected to lyophilization. All peptides (both crude and purified) were stored as lyophilized solids at 4 °C.

### **OPV3** peptide (HO-DFAA-**OPV3**-AAFD-OH)

Prepared according to literature procedure;^42^,^43^ characterization data matches that of the literature. Crude peptide obtained was observed as a yellow powder (*λ*_max_ = 367 nm at pH = 8; HPLC purified, 52.6 mg, 18%). MS (ESI^–^) 1177.7 (M – 1H)^–^ (calc. 1177.46), *m*/*z* 588.3 (M – 2H)^2–^ (calc. 588.2). ^1^H NMR (400 MHz, d_6_-DMSO) *δ*, ppm: 8.51 (d, 1H, 7.3 Hz), 8.03 (d, 1H, *J* = 6.4 Hz), 8.02 (d, 1H, *J* = 7.1 Hz), 7.92 (d, 2H, *J* = 8.4 Hz), 7.71 (t, 3H, *J* = 8.4 Hz), 7.41 (m, 2H), 7.23 (d, 4H, *J* = 4.4 Hz), 7.21–7.13 (m, 1H), 4.50–4.42 (m, 2H), 4.28–4.20 (m, 1H), 3.07 (dd, 1H, *J* = 15.2, 4.3 Hz), 2.82 (dd, 1H, *J* = 16.8, 11.0 Hz), 2.44–2.36 (m, 1H), 1.31 (d, 3H, *J* = 7.2 Hz), 1.21 (d, 3H, *J* = 7.0 Hz).

### **4T** Peptide (HO-DFAA-**4T**-AAFD-OH)

Solid-supported Wang-DFAA-NH_2_ peptide *N*-acylated with 5-bromothiophene-2-carboxylic acid was prepared (0.5 mmol). The peptide was coupled with 5,5′-bis-tributylstannyl-[2,2′]-bithiophene (0.25 mmol, 0.186 g) in the presence of Pd(PPh_3_)_4_ (0.02 mmol, 0.023 g) using the general on-resin Stille coupling procedure. The resin was then subjected to the general cleavage procedure. Crude peptide obtained was observed as an orange powder (*λ*_max_ = 416 nm at pH = 8; HPLC purified, 43.5 mg, 14%). MS (ESI^–^) *m*/*z* 1225.5 (M – 1H)^–^ (calc. 1225.3), *m*/*z* 1247.5 (M – 2H + Na)^–^ (calc. 1247.3), *m*/*z* 1291.5 (M – 4H + 3Na)^–^ (calc. 1291.3), *m*/*z* 612.4 (M – 2H)^2–^ (calc. 612.2), *m*/*z* 311.2 (M – 5H + Na)^4–^ (calc. 311.1) *m*/*z* 325.2 (M – 6H + 2K)^4–^ (calc. 324.6). ^1^H NMR (400 MHz, d_6_-DMSO) *δ*, ppm: 8.62 (d, 1H, *J* = 7.8 Hz), 8.34 (br s, 1H), 8.05 (dd, 2H, *J* = 16.0, 7.9 Hz), 7.86 (d, 1H, *J* = 4.0 Hz), 7.76 (d, 1H, *J* = 6.2 Hz), 7.43 (d, 1H, *J* = 3.8 Hz), 7.38 (d, 2H, *J* = 3.8 Hz), 7.22 (d, 4H, *J* = 4.3 Hz), 7.18–7.14 (m, 1H), 4.45–4.41 (m, 2H), 4.27–4.19 (m, 1H), 4.11–4.03 (m, 1H), 3.06 (dd, 2H, *J* = 14.6, 4.2 Hz), 2.86–2.78 (dd, 2H, *J* = 16.0, 8.7 Hz), 2.44 (m, 1H), 2.36 (dd, 1H, *J* = 15.8, 2.4 Hz), 1.28 (d, 3H, *J* = 7.2 Hz), 1.21 (d, 3H, *J* = 7.1 Hz).

### Reverse phase high-performance liquid chromatography (RP-HPLC)

Peptides that underwent Stille coupling were dialyzed prior to HPLC purification in order to completely remove any excess Pd. The HPLC samples were prepared from lyophilized peptide solids after the dialysis procedure and were dissolved in Milli-Q water as basic samples by adding μL amounts of 1 M KOH until the solution reaches pH 8 to 9. Purification and analysis were performed using an Agilent SD1 PrepStar System with a Phenomenex C8 column (Luna 5 μm, 250 × 21.20 mm and 250 × 4.60 mm). The mobile phase used consists of an ammonium formate aqueous buffer (∼pH 8) and acetonitrile.

### Electrospray ionization mass spectrometry (ESI-MS)

Samples for ESI-MS analyses were prepared in a 1 : 1 methanol and water solution with 1.0% (v/v) ammonium hydroxide. Mass spectra were collected using a Thermo Finnigan LCQ Deca Ion Trap Mass Spectrometer in negative mode.

### Attenuated total reflectionfourier transform infrared (ATR-FTIR) spectroscopy

All data were obtained on dried peptides using a Thermo Scientific Nicolet iD5 ATR-IR. The spectra of coassemblies were taken from the lyophilized acidic peptide solutions.

### Dynamic light scattering (DLS)

All samples were prepared from lyophilized peptide solids and dissolved in Milli-Q water. The pH of the samples was adjusted accordingly (∼pH 2 for acidic samples and ∼pH 10 for basic samples) using 1 M HCl or 1 M KOH. All measurements were recorded using a Zetasizer Nano-ZS90 (Malvern Instruments).

### UV-vis and photoluminescence (PL)

All samples for absorption and emission scans were prepared by dissolving lyophilized peptides in degassed Milli-Q water. The pH of the samples was adjusted accordingly (∼pH 2 for acidic samples and ∼pH 10 for basic samples) using 1 M HCl or 1 M KOH. Acidic and basic samples were separately prepared from a neutral peptide stock solution (*ca.* pH 7; 100 μM), keeping the final concentration for both samples the same and having an optical density of 0.1 to 0.2 for the acidic samples. Reacidified samples were prepared from acidic samples, wherein we added μL amounts of 1 M KOH and mixed (re-basified), then added μL amounts of 1 M HCl and mixed. Thermally annealed samples were prepared from acidic solutions that were heated to 80 °C for 30 min and then cooled down to room temperature. The absorption spectra in the UV-vis region were obtained using a Varian Cary 50 Bio UV-vis spectrophotometer. The photoluminescence spectra were obtained using a PTi Photon S2 Technology International Fluorometer with an Ushio Xenon short arc lamp. All UV-vis and PL spectra reported were recorded within an hour of sample preparation, unless otherwise stated.

### Circular dichroism (CD)

Samples for CD analyses were dissolved in Milli-Q water with pH values adjusted accordingly (∼pH 2 for acidic samples and ∼pH 10 for basic samples) using 1 M HCl or 1 M KOH. The spectra were collected using a Jasco J-810 spectropolarimeter at ∼20 °C (unless otherwise stated), taking the final spectrum from the average of three scans.

### Transmission electron microscopy (TEM)

Peptide nanostructures were imaged by preparing 0.1 wt% (1 mg mL^–1^) peptide solutions in Milli-Q water. Samples were adsorbed for 5 minutes at 25 °C onto 200 mesh copper grids coated with Formvar in carbon film and were stained with a 2% uranyl acetate solution. The grids were allowed to dry prior to imaging. Images were acquired using a Philips EM 420 transmission electron microscope equipped with an SIS Megaview III CCD digital camera, at an accelerating voltage of 100 kV.

## Results and discussion

### Design considerations

**Fig. 1:**
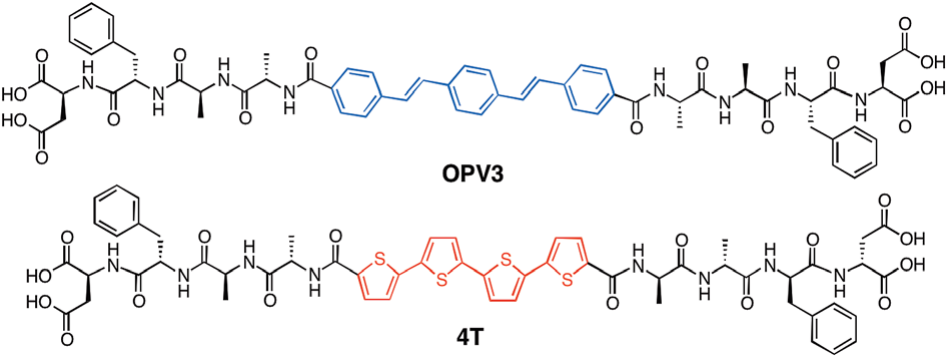
Molecular structures the DFAA-based **OPV3** and **4T** peptides studied herein.

Our system involves two peptide–π–peptide triblock molecules that have pH-dependent assembly, generally resulting in essentially dissolved structures under basic conditions (*ca.* pH 10) and 1D-nanostructures under acidic (*ca.* pH 2) conditions (ESI, Fig. S8 and S9†).^44^,^45^ Two π-conjugated molecules with known semiconducting properties were used as π-electron cores within the triblock system: 1,4-distyrylbenzene (OPV3) as the donor unit, an oligo(*p*-phenylenevinylene) which is known to facilitate efficient exciton migration and has well-studied spectroscopic behavior, and quaterthiophene (4T) as the acceptor unit, which is a known hole-transporting organic semiconductor and low-energy dye. These chromophores comprise the functional π-electron element within the molecules synthesized for this study as depicted in Fig. 1 (**OPV3** and **4T**, respectively). We utilize OPV-based assemblies, which are well-established to have fast exciton diffusion processes and energy transfer processes possible within these types of ordered organic-based nanomaterials.^5^,^12^,^46^,^47^ Energy transfer within OPV nanoparticles doped with quinquethiophene (5T) in films has been reported.^48^

For OPV3 and 4T, both of these chromophores have separately been incorporated into peptide–π–peptide molecules that undergo self-assembly to form nanofibrillar structures with high aspect ratios in aqueous environments.^42^,^43^,^49^,^50^ The particular **OPV3** and **4T** π-conjugated units were chosen because they have comparable molecular lengths and thus can be reasonably expected to encourage intermolecular hydrogen-bonding interactions within a coassembled nanostructure. The aspartic acid–phenylalanine–alanine–alanine (DFAA) tetrapeptide sequence was chosen because the OPV3-assemblies derived from the DFAA peptide sequence were shown previously to generate high-aspect ratio nanostructures with vibronic photoluminescence features associated with high-energy exciton-like emission, making it easier to differentiate the emission of the **OPV3** donor from the **4T** acceptor.^43^,^51^

### 1D-assembly structure/morphology

**Fig. 2:**
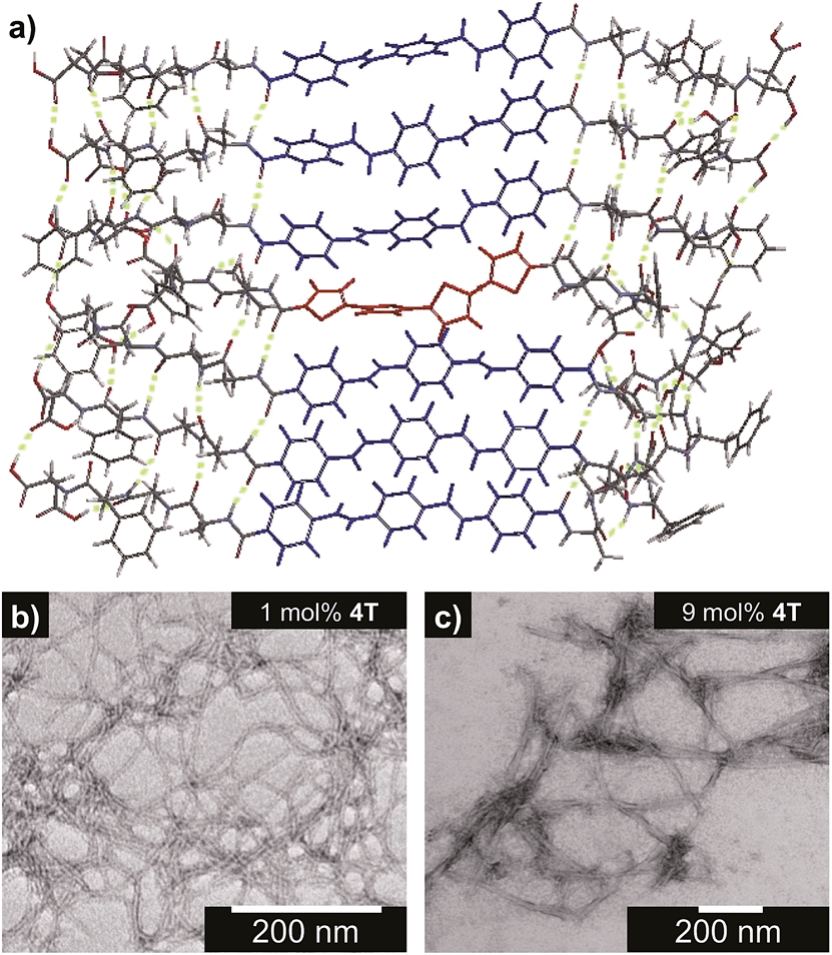
(a) Energy-minimized assembly model for a hypothetical portion of an **OPV3**–**4T** heterostructure with a single isolated **4T** unit within the **OPV3** majority aggregate. TEM images (stained with 2% uranyl acetate) along with the observed widths of nanostructures formed from 0.1 wt% acidic, coassembled **OPV3**–**4T** solutions: (b) 1 mol% (5.6 ± 0.64 nm) and (c) 9 mol% **4T** (13.6 ± 2.3 nm).

Fig. 2a shows an energy-minimized model generated from low-level equilibrium geometry calculations of a portion of 1D-assembly structure whereby the internal hydrogen-bonding networks deviate from ideal β-sheet architectures yet still allow for enthalpic stabilization. Our past molecular dynamics simulations on OPV3-embedded peptides found that the deviations of these assemblies from ideal β-sheet conformations can be attributed to the entropic mixing within the stacks and internal deformations that are brought by a combination of various stabilizing hydrogen-bonding interactions that do not solely rely on strict interpretations β-sheet interactions.^43^ The quadrupole associated with the central π-conjugated structure presents a distinctly non-natural intermolecular interaction that further skews these oligopeptide assemblies from ideal protein secondary structures. This deviation is observed even in pure **OPV3** or **4T** assemblies (ESI, Fig. S10 and 11†), but is more pronounced in the coassemblies (Fig. 2a and ESI, Fig. S12†).

Nevertheless, the generated structural models support the formation of 1D-stacks stabilized by intermolecular hydrogen-bonding interactions that place the donor and acceptor units into intimate intermolecular electronic contact. The IR spectra of assembled **OPV3**, **4T** and mixtures (ESI, Fig. S13†), on the other hand, show the preference for β-sheet-like formation as supported by the presence of Amide I (∼1630 cm^–1^) and Amide II (∼1530 cm^–1^) bands in assembled **OPV3** and even in the coaasemblies (maintained up to 50 mol% **4T**). We used transmission electron microscopy (TEM) to image the morphologies of the nanostructures formed in acidic **OPV3**–**4T** mixtures. Unlike the previously reported high-aspect ratio morphology of **OPV3**, the TEM images of pure **4T** show irregularly-shaped nanostructures under acidic conditions (ESI, Fig. S14†). However, TEM indicates that the addition of acceptor **4T** did not significantly perturb the self-assembling ability of the donor **OPV3** as shown by the 1D-nanostructure formation observed at different coassembly ratios up to 50 mol% **4T**, which corroborates with the IR spectra. The only obvious difference was the change in the widths of each nanostructure as the **4T** component was increased, suggesting a change in the bundling behavior within each nanostructure upon coassembly (Fig. 2b–c and ESI, Fig. S15 and 16†). The bundling behavior, which relies on the lateral interactions between each “1D-stack”, depends on the local microenvironment of the assembly which are often challenging to control even for naturally occurring amyloid-forming peptides.

### Steady-state photophysical characterization

**Fig. 3:**
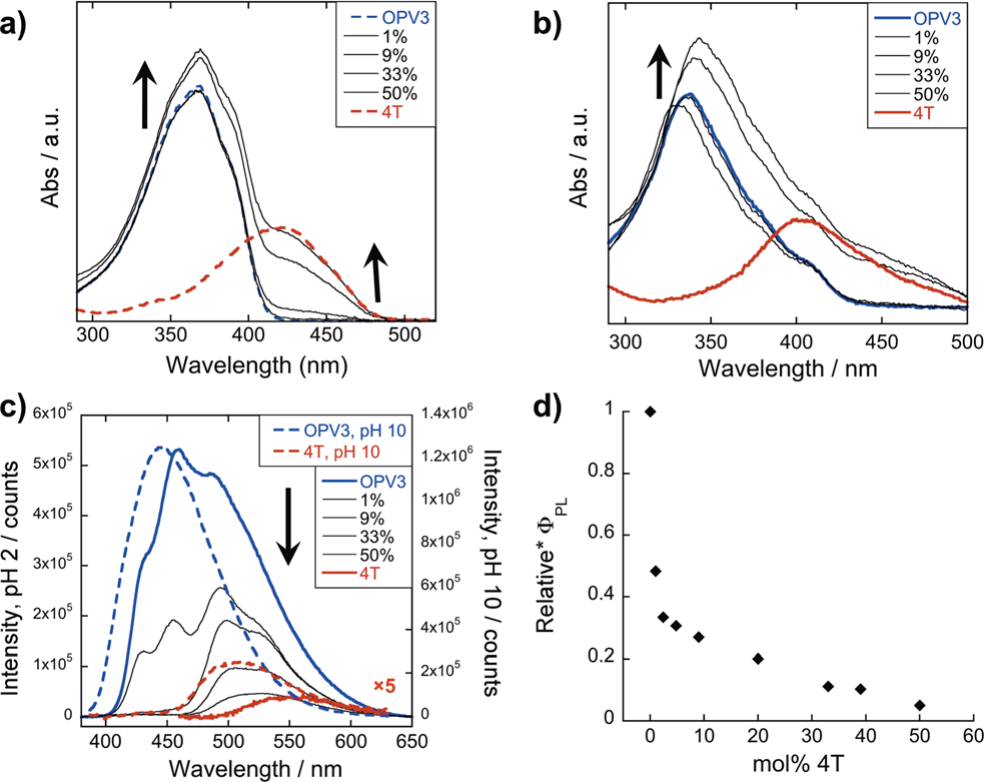
Absorption spectra of **OPV3**, **4T**, and their co-mixtures under basic (a) and acidic (b) conditions. Emission spectra (c) recorded at pH = 10 (---, *λ*_exc_ = 320 nm) and pH = 2 (—, *λ*_exc_ = 330 nm). For comparison, a direct excitation of **4T** molecules and assemblies at 450 nm is shown in (c). [**OPV3**] = 3.2 μM, [**4T**] = 0.03–3.2 μM; arrows show trends as mol% **4T** is increased. Quantum yields (d) of the **OPV3**–**4T** mixtures at pH = 2 (*λ*_exc_ = 330 nm), relative to **OPV3**.

The steady-state absorption and photoluminescence (PL) spectra of the donor and acceptor solutions (both dissolved and assembled) were recorded as baseline points (Fig. 3a–c). Under basic (dissolved) conditions, **OPV3** has an absorption *λ*_max_ at 367 nm and emission *λ*_max_ at 448 nm while **4T** absorbs at 416 nm and emits at 510 nm. Upon initiating assembly under acidic conditions, both **OPV3** and **4T** show a blue-shift in absorbance with respect to their basic counterparts, having *λ*_max_ at 339 and 400 nm, respectively. A broad, featureless emission peak with a *λ*_max_ at 561 nm was observed for the assembled **4T** solution upon direct excitation at 450 nm. For the assembled **OPV3** solution, vibronic features suggesting an excitonic emission were observed, with distinct peaks appearing at 431, 460 and 490 nm. These steady-state spectral properties are consistent with those found previously for **OPV3**^43^ and for a peptide sequence variant of **4T**,^40^,^49^ with the blue-shift in absorption and PL quenching and red-shift observed in acidic samples indicating the formation of “H-like” aggregates. The differences in PL inherent to these two chromophores allow us to distinguish spectral signatures originating from the **OPV3** donor and the **4T** acceptor.

As for the coassembled heterostructures, UV-vis and PL spectra of the **OPV3**–**4T** mixtures were recorded during titration experiments where **OPV3** concentration was held constant ([**OPV3**] = 3.2 μM) in order to monitor the photophysical events during the coassembly process. The emission spectra of the mixtures shown in Fig. 3c correspond to solutions that were excited at 320 nm (basic) and 330 nm (acidic), the wavelengths at which **OPV3** has reasonable absorption but **4T** has minimum absorption. The absorption profile consistently shows a blue-shifted absorption of acidic against the basic solutions in all mol% **4T**, indicating the fidelity of the “H-like” aggregation (Fig. 3b). In the dissolved state (*ca.* pH 10), discrete spectral peaks for **OPV3** and **4T** in the absorption spectra (Fig. 3a) and peaks characteristic of dissolved **OPV3** in the emission spectra (ESI, Fig. S17a†) were observed for mixtures of **OPV3** and **4T** wherein [**OPV3**] was kept constant. When the overall chromophore concentration is kept constant (ESI, Fig. S18b and c†), discrete spectral peaks for **OPV3** and **4T** emission were observed for the basic solutions, further illustrating the absence of interactions (*e.g.*, collisional quenching) and energy transfer between the donor–acceptor pair in their dissolved state. For the acidic mixtures, where nanostructure formation is expected to occur, the emission peaks characteristic of the donor **OPV3** became quenched along with the decrease in relative quantum yield (Fig. 3d) upon the addition of the acceptor **4T**, giving rise to a new spectral feature (*λ*_em_ ∼ 540 nm) reminiscent of **4T** emission. The progressively red-shifted PL spectral features of the coassembled solutions as the acceptor concentration increases are also indicative of energy transfer. The donor peak quenching was observed even by adding 1 mol% of the acceptor **4T**, leading to the decrease in the PL peak area down to 47%. This suggests that during the lifetime of the **OPV3** excited state, a significant fraction of the photogenerated excitons has the ability to migrate to the **4T** acceptor dopant. In an analogous study by Adams and coworkers, where donor and acceptor π-systems were conjugated to dipeptides within a hydrogel, only a 15% decrease in the emission intensity of the donor at 0.8 mol% acceptor was observed.^36^ Nalluri and Ulijn were able to increase the efficiency by using a related dipeptide that formed a gelator system using an enzymatic assembly trigger and observed complete quenching of donor PL at an acceptor concentration of about 3 mol%.^37^ Both of these examples demonstrated energy transfer using end-functionalized peptide hydrogelators with alkoxy naphthalene donor combined with a dansyl derivative acceptor whereas here, we present a peptide-based donor–acceptor pair with a comparable energy transfer efficiency occurring between the semiconductor units that are buried within peptide–π–peptide nanostructures.

At 9 mol% **4T**, complete quenching of the higher energy vibronic features characteristic to aggregated **OPV3** emission peak was observed. In parallel studies involving more hydrophobic peptide sequences attached to the quaterthiophene core, we have found similar PL profiles under acidic, self-assembling conditions as observed with the 9 mol% **4T** acidic solution (ESI, Fig. S19†). The broad, featureless peak observed from 33 to 50 mol% **4T** (Fig. 3c and ESI, Fig. S17b†) exhibits a red shift as the mol% **4T** increases within this range, and can be attributed to the contribution of **4T** emission due to the absence of spectral features characteristic of assembled **OPV3**. This broad peak is an apparent superposition of the emission *λ*_max_ peaks associated with dissolved (510 nm) and aggregated (561 nm) **4T**, and suggests the coexistence of “isolated” and “aggregated” **4T** exciton traps diluted within the aggregated **OPV3** π-stacked nanostructures.

**Fig. 4:**
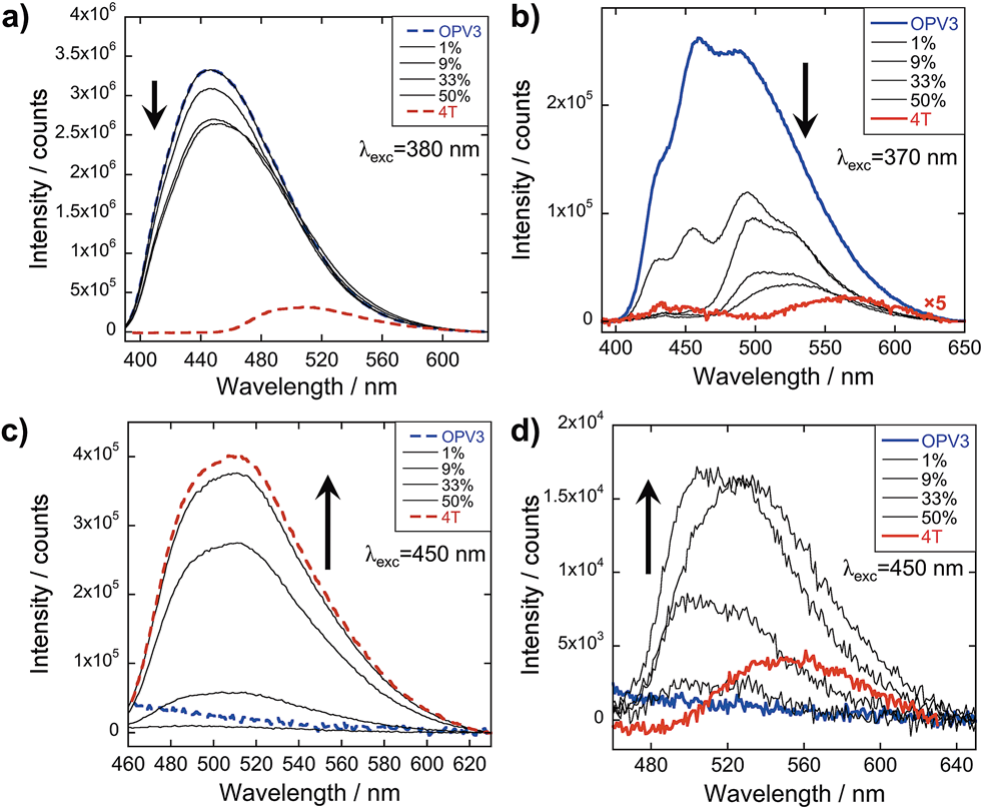
PL spectra for **OPV3** and **4T** coassemblies (arrows indicate increasing mol% **4T**) at different excitation wavelengths; solutions in (a) and (c) are at pH = 10 (---), (b) and (d) at pH = 2 (—).

The emission spectra were then recorded after excitation at other wavelengths (Fig. 4 and ESI, Fig. S20†): one at a higher energy wherein both **OPV3** and **4T** strongly absorbs (380 nm for basic and 370 nm for acidic) and one at a lower energy wherein **4T** absorbs but **OPV3** does not (450 nm). With the higher energy excitation, the resulting emission spectral features of both the basic and acidic solutions are similar to that excited at 320 or 330 nm, only varying in intensities due to the higher extinction coefficient of the system at the latter longer wavelengths. The extent of quenching at 1 mol% **4T** was also similar (peak area decreased down to 47%) when the solutions are excited at 320 and 330 nm.

On the other hand, having the excitation at 450 nm, wherein only **4T** strongly absorbs, the resulting emission profiles for the basic solutions only correspond to **4T**. The PL peaks for the coassemblies are blue-shifted with respect to pure **4T** emission that is possibly due to the less planar **4T** conformation when locked within the stacks of **OPV3** than within **4T** units as observed in the energy-minimized models. Note that at 9 mol% **4T**, the PL intensity when the acidic solution is excited at 330 nm is about 25-fold higher than when the **4T** is directly excited at 450 nm—confirming the contributions of energy transfer from **OPV3** to **4T** in the coassembled nanostructures.

**Fig. 5:**
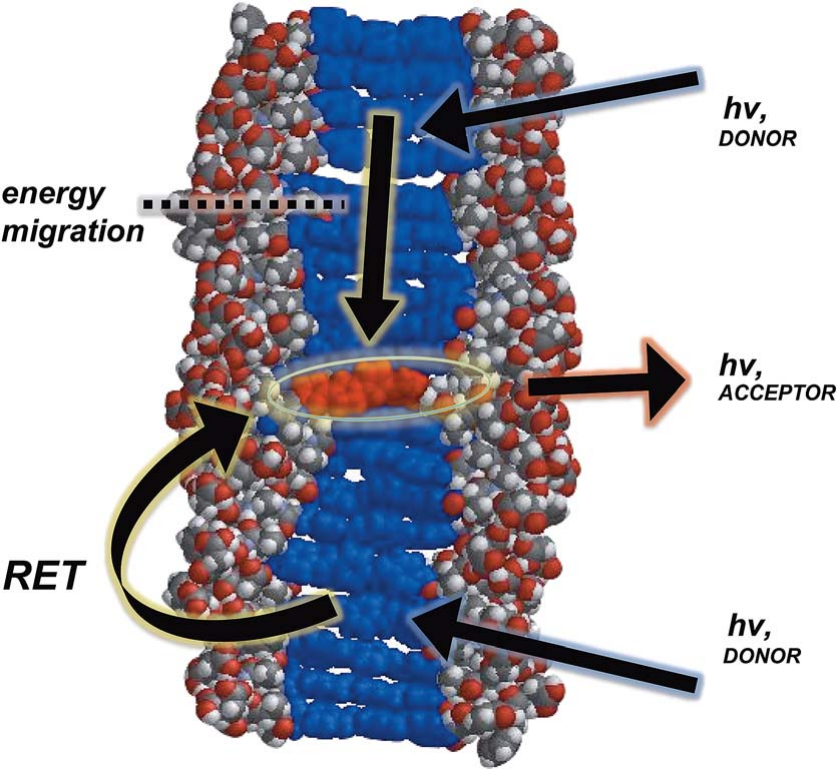
Space-filling, energy-minimized model of **4T** coassembled within 1D-stacks of **OPV3**, illustrating the possible energy transfer processes (resonance energy transfer (RET) and carrier migration).

In general, a Förster-like energy transfer that is governed by dipole–dipole interactions requires the donor–acceptor system to have strong spectral overlap between the donor emission and acceptor absorbance. Such requirement is achieved in both the dissolved and aggregated states of **OPV3** and **4T** donor–acceptor pair (ESI, Fig. S21†). These nanomaterials can be envisioned as ensembles of different local intermolecular chromophore distances and dipole orientations,^43^ so extracting specific geometric information or otherwise modelling Förster-type processes would be unreliable. The chosen donor–acceptor pair can foster a direct resonance energy transfer, given the spectral overlap, but the significant changes in the emission spectral features observed even at very low loadings of the **4T** acceptor within the coassemblies and the fact that OPV-based stacks are known to facilitate fast exciton migration implies that other mechanisms are potentially involved for the overall transport process (Fig. 5). In addition, increasing the proximity between donor–acceptor units by trapping them within the 1D-nanostructures potentially allows shorter-range energy transfer processes that result from direct wavefunction overlap between the interacting chromophores to occur, which are generally known to increase transport within a chain of conjugated polymers as compared to those solely dictated by dipole–dipole interactions.^52^

### Lifetime measurements

To further support that the spectral changes observed above are consequences of the funneling of energy from **OPV3** to **4T***via* exciton migration, fluorescence lifetime measurements were recorded at different **OPV3** and **4T** mixture ratios (Table 1; ESI, Fig. S22 and S23†). The PL decay profiles for pure **OPV3** and **4T** assemblies as well as their coassemblies can be fit to a two-component exponential while a single component fit was suitable for the decay profiles of basic solutions. The drastic decrease in the average lifetime starting at 1 mol% **4T** at the excitation wavelengths where **OPV3** only significantly absorbs and where **4T** has minor absorbance contributions (340 and 375 nm, respectively), similar to the observation in energy-transporting OPV gels studied by Ajayaghosh and coworkers,^7^ and the progressively decreasing fraction of the long-lived component upon increasing the mol% of the acceptor under acidic conditions supports the energy transfer within the coassemblies.

**Table 1:** Fluorescence lifetimes of **OPV3**, **4T**, and their coassemblies^*a*^ in acidic (pH 2; *λ*_exc_ = 340, 375 nm; *λ*_em_ = 490, 510 nm) and basic (pH 10; *λ*_exc_ = 375 nm; *λ*_em_ = 510 nm) solutions

Mol% **4T**	*τ*/ns				
	pH 10	pH 2, *λ*_exc_ = 375 nm		pH 2, *λ*_exc_ = 340 nm	
		*τ* (%)	*τ*_avg_	*τ* (%)	*τ*_avg_
0%	1.06	1.10 (69.8); 5.28 (30.2)	2.03	1.08 (64.1); 5.06 (35.9)	2.51
1%	1.06	0.968 (78.6); 4.84 (21.4)	1.79	0.752 (80.5); 4.48 (19.5)	1.48
5%	1.06	0.767 (94.6); 3.39 (5.44)	0.91	0.748 (91.7); 5.72 (8.33)	1.16
13%	1.05	0.720 (98.0); 6.71 (2.00)	0.84	0.663 (97.9); 5.89 (2.05)	0.77
26%	1.06	0.664 (99.2); 4.13 (0.78)	0.69	0.610 (97.5); 4.85 (2.48)	0.72^*b*^
33%	1.06	0.631 (99.6); 4.02 (0.36)	0.64	0.614 (98.6); 4.81 (1.39)	0.67^*b*^
100%	0.55	0.528 (82.0); 1.67 (18.0)	0.73	N/A^*c*^	N/A^*c*^

^*a*^Full decay traces can be found in ESI, Fig. S22 and S23.

^*b*^Emission recorded at 510 nm where PL intensities are higher for these data points.

^*c*^Insufficient intensities observed for meaningful measurements.

This also supports the minimal contributions from **OPV3** excimer formation, which would result in a low-energy charge transfer band that would overlap with the **4T** emission profile but has a longer-lived lifetime.^43^,^53^ In addition, the absence of energy transfer in the dissolved solutions and the need for nanostructure formation to initiate the energy transfer within an ordered matrix, which is also found important in the systems studied by Meijer and coworkers,^5^,^12^ is further verified by the unchanging lifetime decay values of the **OPV3**–**4T** basic mixtures with respect to pure, basic **OPV3** solution. It will be interesting to further study the exciton dynamics within these assemblies, however, OPV nanostructures are known to exhibit ultrafast exciton migration dynamics faster than what can be captured by the time-resolved measurements performed herein. Future work will further probe the specifics of the migration^46^*via* ultrafast anisotropy decay and transient absorption measurements.

### Effect of 1D-heterostructure formation on the energy transfer efficiency

**Fig. 6:**
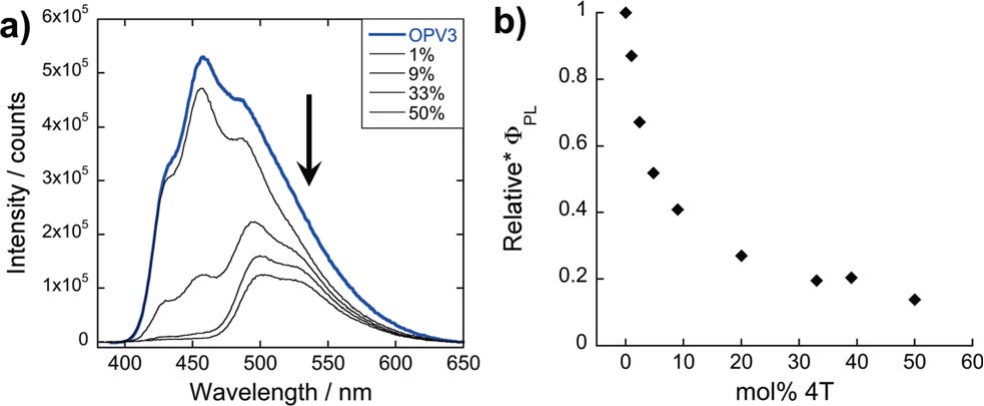
(a) PL spectra (*λ*_exc_ = 330 nm) of resulting solutions from titrating **4T** to a pre-assembled **OPV3** acidic solution (arrows indicate increasing mol% **4T**) and (b) their corresponding quantum yields relative to **OPV3**.

The potential mechanisms of energy transfer proposed for this system involve exciton migration along intimately stacked donor chromophores within a 1D-assembly that is trapped by the acceptor. In order to further investigate how this nanoscale ordering improves energy transfer, we monitored the emission spectra of donor–acceptor mixtures wherein a pre-assembled **OPV3** was separately prepared as an acidic solution and then titrated against neutral **4T** (Fig. 6). The TEM images for a 1 : 1 coassembled **OPV3**–**4T** mixture under this condition (ESI, Fig. S16b†) shows that this forms two different aggregate morphologies that are indicative of the favored separate aggregation of **OPV3** and **4T**, potentially generating nanomaterial interfaces similar to p–n heterojunctions^54^,^55^ for the semiconductor chromophores.

Unlike the coassemblies reported upon in Fig. 3 and 4 prepared by acidification of dissolved solutions of **OPV3** and **4T**, wherein **4T** can serve as a local trap for diffusing excitons along the 1D-nanostructures, assemblies formed from the solution wherein **4T** was titrated with a pre-assembled **OPV3** is expected to foster an energy transfer process that would mostly rely on non-specific dipole interactions or diffusive encounters. Supramolecular assemblies are presumed to be dynamic in nature, so it is possible to envision free **4T** molecules being able to intercalate within a pre-assembled **OPV3** nanostructure. However, it is expected that the **4T** molecules should be primarily driven to assemble under these solution conditions into segregated nanostructures, as supported by the TEM observations. At 1 mol% **4T**, the peak area of the emission profile of assemblies (Fig. 6a) generated from this condition only decreased down to 82% with respect to pure **OPV3**, showing a much less efficient energy transfer than when the **OPV3** and **4T** are intimately coassembled. Moreover, the complete quenching of the donor vibronic features at ∼430 and 460 nm, which was observed at 9 mol% for the coassembled mixtures, was only observed starting at 33 mol% **4T** (ESI, Fig. S24†) when **4T** is titrated against pre-assembled **OPV3** nanostructures.

### Reversibility of heterostructure formation

We also explored the dynamic nature of these coassembled nanostructures when exposed to different environmental stimuli such as pH and temperature. These conditions would allow us to investigate different kinetic regimes for nanostructure formation beyond the kinetically-trapped state obtained upon initial solution acidification. The pH-dependence of nanostructure formation for these π-conjugated peptides is already well-established, but its reversibility and the underlying consequences of pH switching to the energy transfer within our peptide heterostructure system are not yet explored. Similarly, an effort was also made to explore the thermal response of both nanostructure formation and efficiency of energy transfer due to the temperature-dependent assembly mechanisms existing in the related energy-transporting oligo(*p*-phenylenevinylene)-based systems studied by Meijer and coworkers.^5^,^12^

#### Variation of pH

**Fig. 7:**
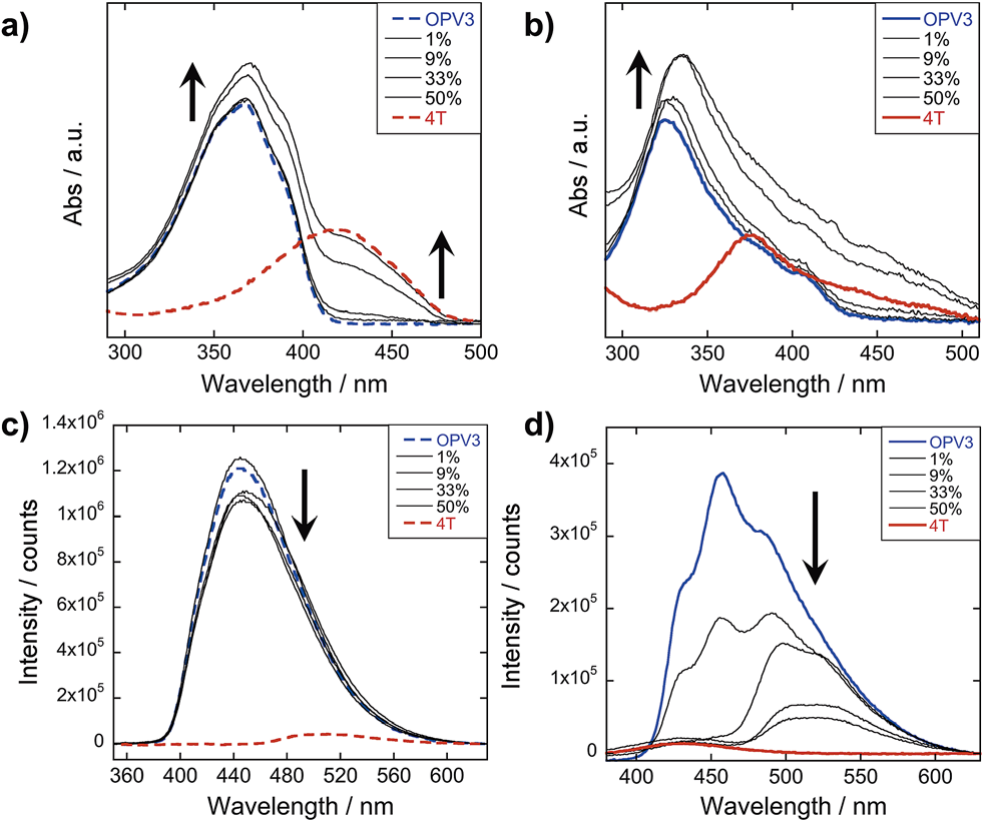
UV-vis (a and b) and PL (c and d) spectra of (a and c) re-basified (*λ*_exc_ = 320 nm) and (b and d) reacidified solutions used in Fig. 3a and c (*λ*_exc_ = 330 nm); pH = 10 (---), pH = 2 (—); arrows indicate increasing mol% **4T**.

Interestingly, energy transfer was observed to be reversible within a pH-controlled assembly-dissolution cycle (Fig. 7). From the acidic coassembled samples, we tested whether we could use pH as a “switch” that could turn on or off the occurrence of energy transfer within the assemblies. Upon adding base, the solution goes back to the dissolved state that does not demonstrate energy transfer as confirmed by the absorption and emission spectra (Fig. 7a and c).

After reacidifying these samples, quenching of the **OPV3** donor emission peaks, along with the development of the new spectral peaks reminiscent of **4T** emission, suggests that the re-assembled structure still fosters the energy transfer process (Fig. 7b and d). Comparing these to the emission spectra shown in Fig. 3c, an enhancement in the intensity in the central vibronic feature at 455 nm relative to 430 and 490 nm shoulder intensities was observed. However, the intensity of this 455 nm decreased by ∼25% as compared to the 460 nm peak of the initial acidic **OPV3**, which can be attributed to environmental degradation. Note that the nearly complete quenching of the 430 and 455 nm peaks still occurred at 9 mol% **4T**. The observed trends in the changes of the emission signals were also the same as that of the initial acidic solution when excited at 380 and 450 nm (ESI, Fig. S25†). These observations show an important characteristic of these 1D-nanostructures that even though kinetically-trapped states are formed from rapid re(acidification), the ensemble photophysics remain reproducible.

#### Variation of temperature

In general, an efficient energy transfer occurs in a state wherein the assembled structure of the donor–acceptor pair represents a local thermodynamic minimum within the free energy landscape.^37^,^56^ By subjecting the acidic samples to a heating–cooling cycle (25 °C to 80 °C to 25 °C) as a form of annealing, we could access thermodynamic minima that are potentially different from the initial trapped state formed after rapid sample acidification. The emission spectra of the thermally annealed, acidic samples (ESI, Fig. S26†) were observed to have vibronic features similar to the reacidified sample in Fig. 7d. The existence of quenched vibronic features at 430 and 460 nm observed for the annealed 9 mol% **4T** solution, which deviates from the observed complete quenching of these signals for the initial 9 mol% **4T** acidic samples at 25 °C in Fig. 3c, suggests that the annealed aggregate has an assembly structure that results in excitons being more easily trapped within the donor matrix rather than reaching the acceptor site—leading to the appearance of donor emission features even up to 25 mol% **4T**. It is possible that exposure at high temperatures and subsequent cooling leads to more local disorder at the donor–acceptor interface, thus allowing for more excitons to be trapped within the donor matrix. The PL spectra taken for “aged” acidic samples after *ca.* 16 hours of initial preparation (Fig. S27†) resembled the spectral features observed for annealed samples, supporting that there are other temporally accessible thermodynamic minima beyond the initial trapped state formed by rapid acidification, as would be anticipated for any type of oligopeptide self-assembly platform. These different assembly minima have different energy transfer dynamics which we plan to explore in our future work.

**Fig. 8:**
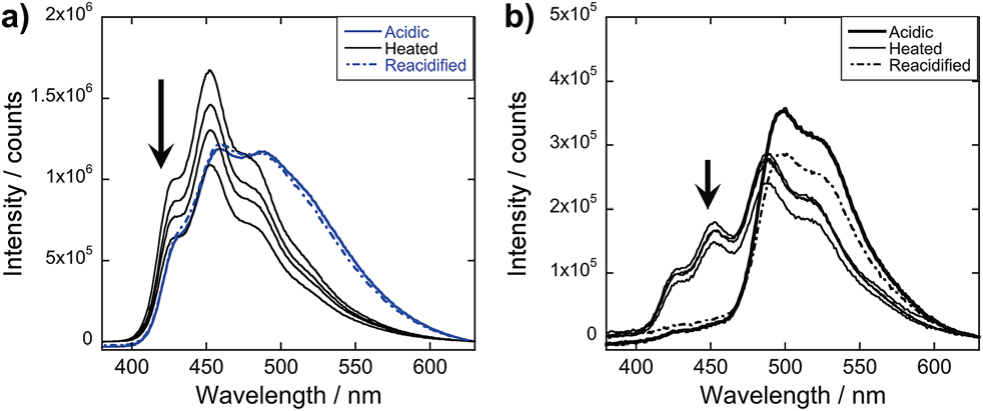
Monitoring the emission *λ*_exc_ = 330 nm during the thermal cycling process for acidic samples of (a) **OPV3** and (b) 9 mol% **4T** solutions; acidic samples (bold line) were heated to 80 °C for 30 minutes, readings taken at *ca.* 60–70 °C and then cooled back to room temperature (thin line); arrows indicate increasing time of cooling within 4 hours; base was then added to these annealed samples and were then reacidified (broken line); [**OPV3**] = 3.2 μM.

To obtain more information about the assembly dynamics and energy transfer as the temperature increases, we took the emission spectra of high temperature samples (*ca.* 60–70 °C) for **OPV3**, **4T**, and 9 mol% **4T** coassembly and monitored the signals as the solutions cool down (Fig. 8 and ESI, Fig. S28†). At a high temperature, the emission signature is still reminiscent of an exciton-coupled structure instead of the dissolved state reported in previous studies of oligo(*p*-phenylenevinylene)-based systems at higher temperatures in organic solvents.^5^ At 9 mol% **4T**, wherein we started to observe the complete quenching of donor emission for the acidic samples, quenched signals were also observed for the heated samples but the ∼430 and 460 nm peaks associated with the **OPV3** vibronic couplings were still present. This indicates that energy transfer is still facilitated in the potentially new aggregate structure formed by heating, however, the efficiency of energy transfer decreased. Moreover, the PL spectra of both **OPV3** and 9 mol% **4T** annealed solutions both showed blue-shifted signals (∼10 nm) as compared to the acidic samples at 25 °C, which is maintained throughout the cooling period. These observations reflect the resistance of these peptide-bound π-systems towards nanostructure disassembly in aqueous environments, even at biologically “extreme” temperatures. The unchanging emission profile upon cooling is also consistent with the recent report by Stupp and coworkers about the hysteresis effect upon heating and cooling back their peptide amphiphiles under different solvent conditions.^57^ It is also interesting to note that following the addition of base to the annealed samples and subsequently reacidifying, the signal recovers back to the initial acidic emission profile. The persistence of self-assembled structures under extreme conditions, as shown by the system studied herein, is important because it is generally addressed that the development of supramolecular polymers that do not break apart at high temperatures remains as a challenge in the field.^58^

**Fig. 9:**
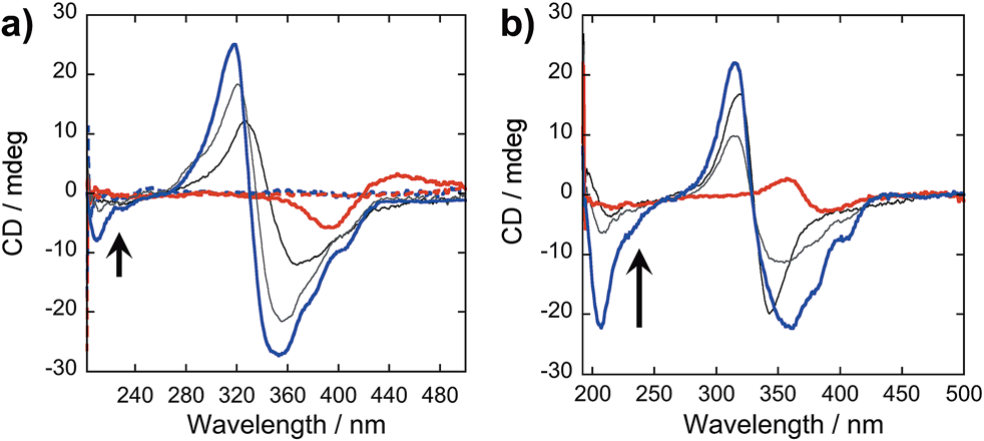
CD spectra of (a) acidic (—; pH 2), basic (---; pH 10), and (b) annealed **OPV3**–**4T** coassembled solutions [**OPV3**] = 3.2 μM; arrows indicate increasing mol% **4T** (4.8%, 50% to 100%).

In our peptide–π–peptide triblock system, we can attribute this structure stability to the contributions of the hydrogen-bonding networks among the peptide segments. The CD spectrum (Fig. 9a) of pure **OPV3** under acidic conditions shows a large negative Cotton effect and a high-energy minimum at ∼220 nm, indicative of β-sheet formation of the peptide moiety that corroborates with the ATR-IR data. On the other hand, the CD spectrum of pure **4T** under acidic conditions shows weak Cotton effect with no signals in the high-energy region. The CD spectra of the dissolved basic samples showed no low energy features, but a bisignate Cotton effect within the chromophore absorption region for the coassembled acidic samples was observed at all mol% **4T**, indicating the existence of exciton-coupled chromophores held within a chiral environment. This supports that the occurrence of 1D-stacking of π-units in a chiral environment is maintained during the coassembly process. Upon annealing the acidic solutions, the high-energy signature indicative of β-sheet formation in the CD spectra for acidic **OPV3** and the coassemblies (Fig. 9b) became more pronounced. Since the energy-minimized structures previously discussed do not show an ideal β-sheet assembly, the enhanced high-energy spectral signature in CD supports the possibility that heating of the 1D-nanostructures leads to a more thermodynamically favored “β-sheet-like” motif that subsequently alters the spatial orientation of the transition dipoles of the chromophores within the aggregate, and thus, their corresponding photophysics as observed in Fig. 8.

## Conclusions

We have demonstrated energy transport within a dynamic donor–acceptor system based on π-conjugated peptide nanostructures in completely aqueous environments. This system shows an energy transfer process that involves multiple mechanisms such as exciton migration and resonance energy transfer. This study also supports the importance of nanoscale order to obtain an efficient energy transport as mediated by the funneling of excitons to the acceptor sites and how these processes are impacted by thermal annealing and repetitive changes in pH. The acquired photophysical data reveal that the nanostructures do not result in complete dissolution upon increasing the temperature to ∼70 °C but instead reflects the formation of another rearranged structure that exhibits hysteresis—indicating nanostructure stability. We can attribute these interesting assembly features to the dynamic and stable nature of hydrogen bonds and side chain interactions of the peptide segments that stabilizes the anisotropic π–π interactions between the monomers. Although the heterostructure reversibility provides insights to the dependence of energy transfer efficiency into appropriately assembled donor–acceptor pair and the stability of this system, a more accurate elucidation of energy transfer mechanism within the annealed 1D-heterostructures requires further investigation of the temperature-dependent nanostructure morphology changes. Overall, this strategy to design bioelectronic materials that utilize peptide interactions to enhance chromophore interactions and energy transfer efficiency is applicable to a range of emerging bio-relevant optoelectronic devices such as artificial photonic antenna systems and other photosynthetic unit mimics.

## Supplementary Material

Supplementary information
