# Supplementary material for: Energy transfer within responsive pi-conjugated coassembled peptide-based nanostructures in aqueous environments
Source: Chem Sci. 2014 Dec 5;6(2):1474–84. doi: 10.1039/c4sc03122a (PMC5811113; doi:10.1039/c4sc03122a)
Supplement: Supplementary file 1 [file SC-006-C4SC03122A-s001.pdf]

Electronic Supplementary Material (ESI) for Chemical Science.
This journal is © The Royal Society of Chemistry 2014

Supporting Information

Energy transfer within responsive pi-conjugated coassembled peptide-based nanostructures in aqueous environments

Herdeline Ann M. Ardoña [a,c] and John D. Tovar [a,b,c]

---

[a] Department of Chemistry, Krieger School of Arts and Sciences, Johns Hopkins University, 3400 N. Charles St., Baltimore, MD 21218 USA;

[b] Institute for NanoBioTechnology, Whiting School of Engineering, Johns Hopkins University, 3400 N. Charles St., Baltimore, MD 21218 USA

[c] Department of Materials Science and Engineering, Whiting School of Engineering; Johns Hopkins University, 3400 N. Charles St., Baltimore, MD 21218 USA

Email: tovar@jhu.edu; *Web*: http://pages.jh.edu/chem/tovar; *Tel*: +14105166065

CONTENTS

Characterization data
- ^{1}H NMR spectra………………………………………………………… S2
- ESI spectra/ Analytical HPLC traces………………………………………… S3-4
- ATR-IR spectra………………………………………………………….. S5

Supplementary data
- DLS data…………………………………………………………………… S6-7
- Energy-minimized models…………………………………………………..S8
- ATR-IR data of lyophilized acidic solutions……………………………….. S9
- Supplementary TEM images………………………………………………...S10
- Supplementary absorption and emission spectra……………………………S11-15
- Fluorescence lifetime decay profiles………………………………………….. S13

CHARACTERIZATION DATA

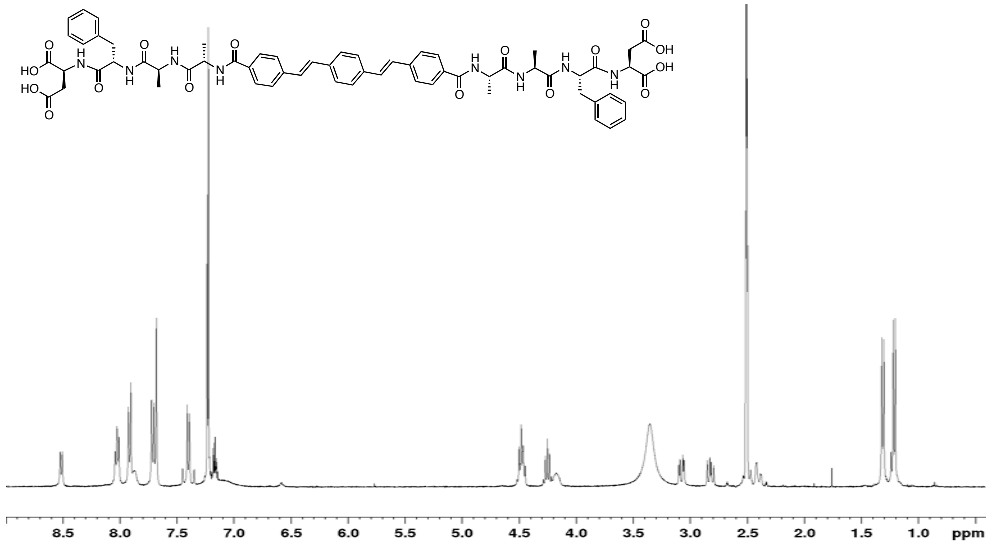

Fig. S1. ^{1}H (400 MHz, DMSO) NMR of HO-(DFAA)$_2$-**OPV3** peptide.

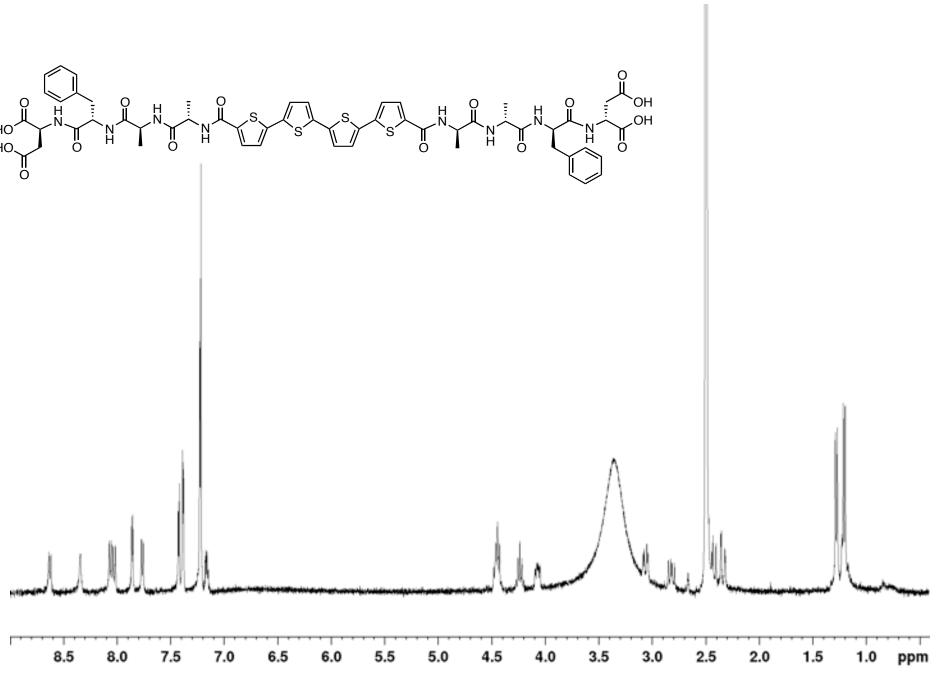

Fig. S2. ^{1}H (400 MHz, DMSO) NMR of HO-(DFAA)$_2$-**4T** peptide.

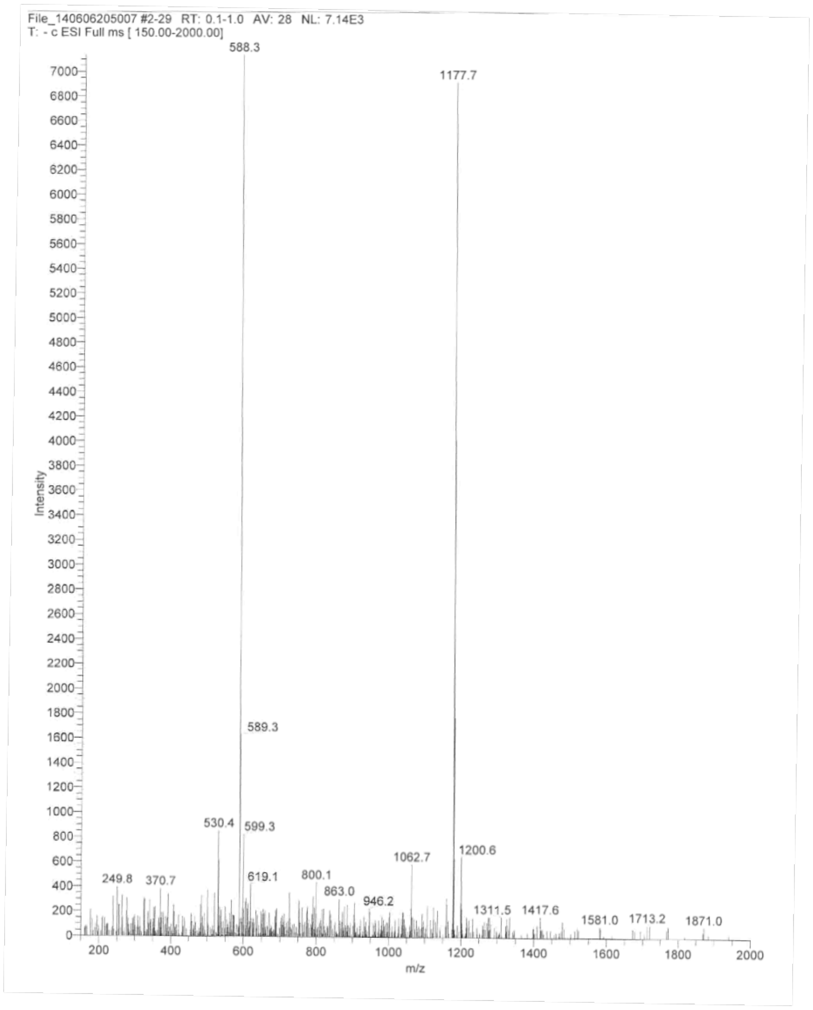

Fig. S3. ESI of HO-(DFAA)$_2$-**OPV3** peptide.

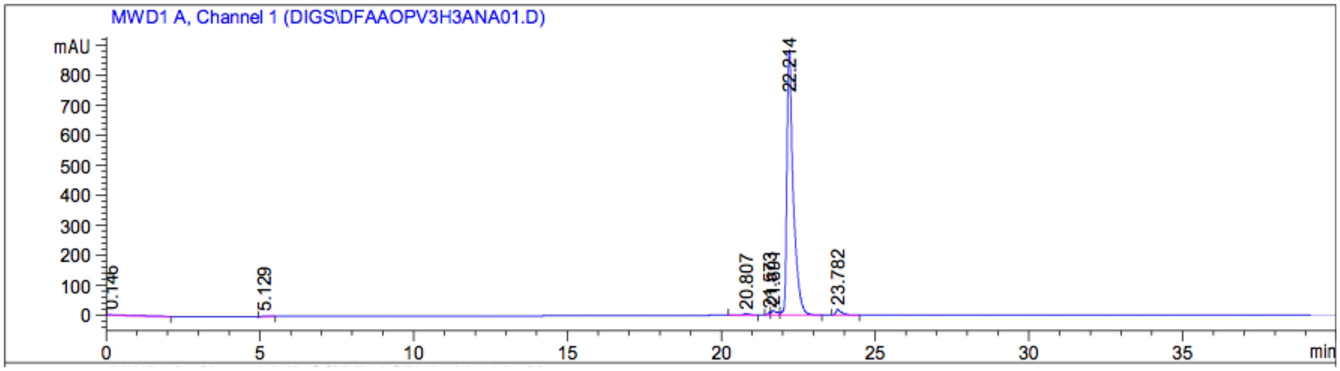

Fig. S4. Analytical HPLC trace of HO-(DFAA)$_2$-**OPV3**, monitoring at 365 nm.

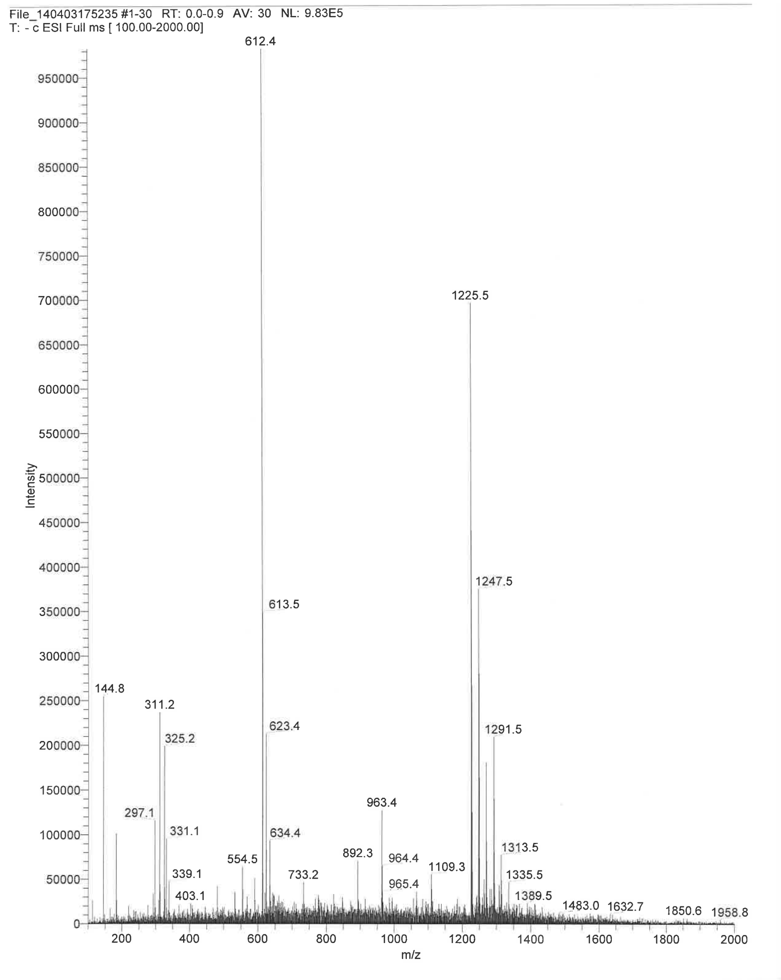

Fig. S5. ESI of HO-(DFAA)$_2$-**4T** peptide.

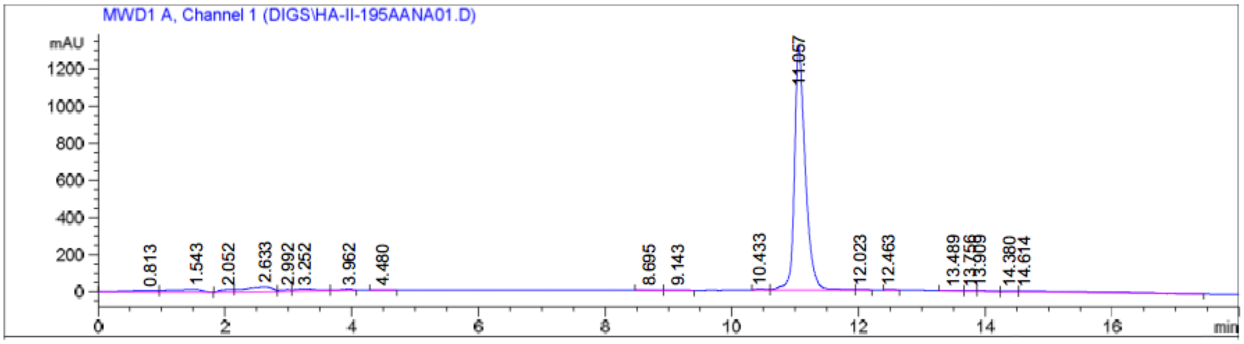

Fig. S6. Analytical HPLC trace of HO-(DFAA)$_2$-**4T**, monitoring at 400 nm.

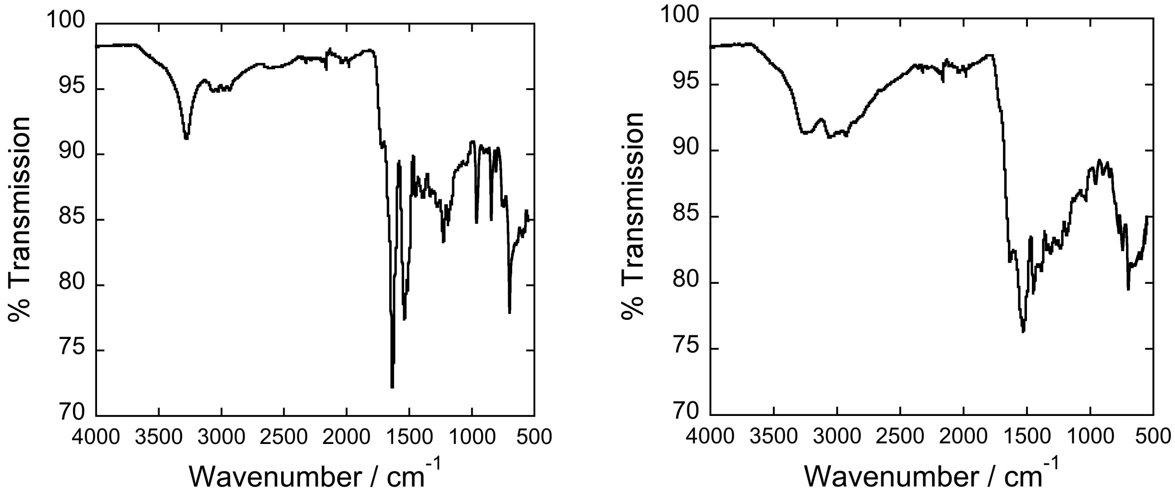

Fig. S7. Attenuated total reflectance IR spectra of **OPV3** (*left*) and **4T** (*right*) in solid state.

SUPPLEMENTARY DATA

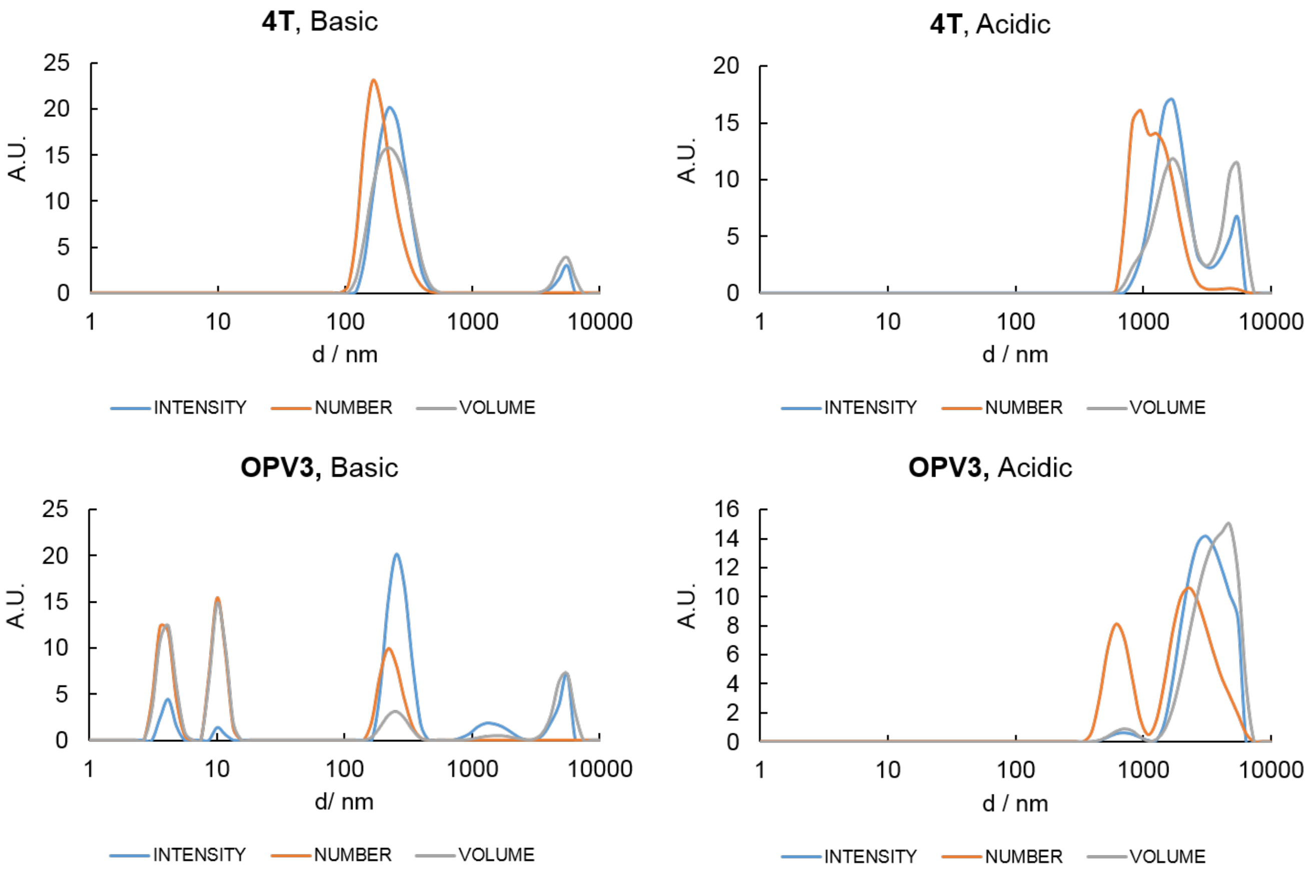

Fig. S8. Dynamic light scattering data for 50μM basic and acidic solutions of **OPV3** and **4T.**

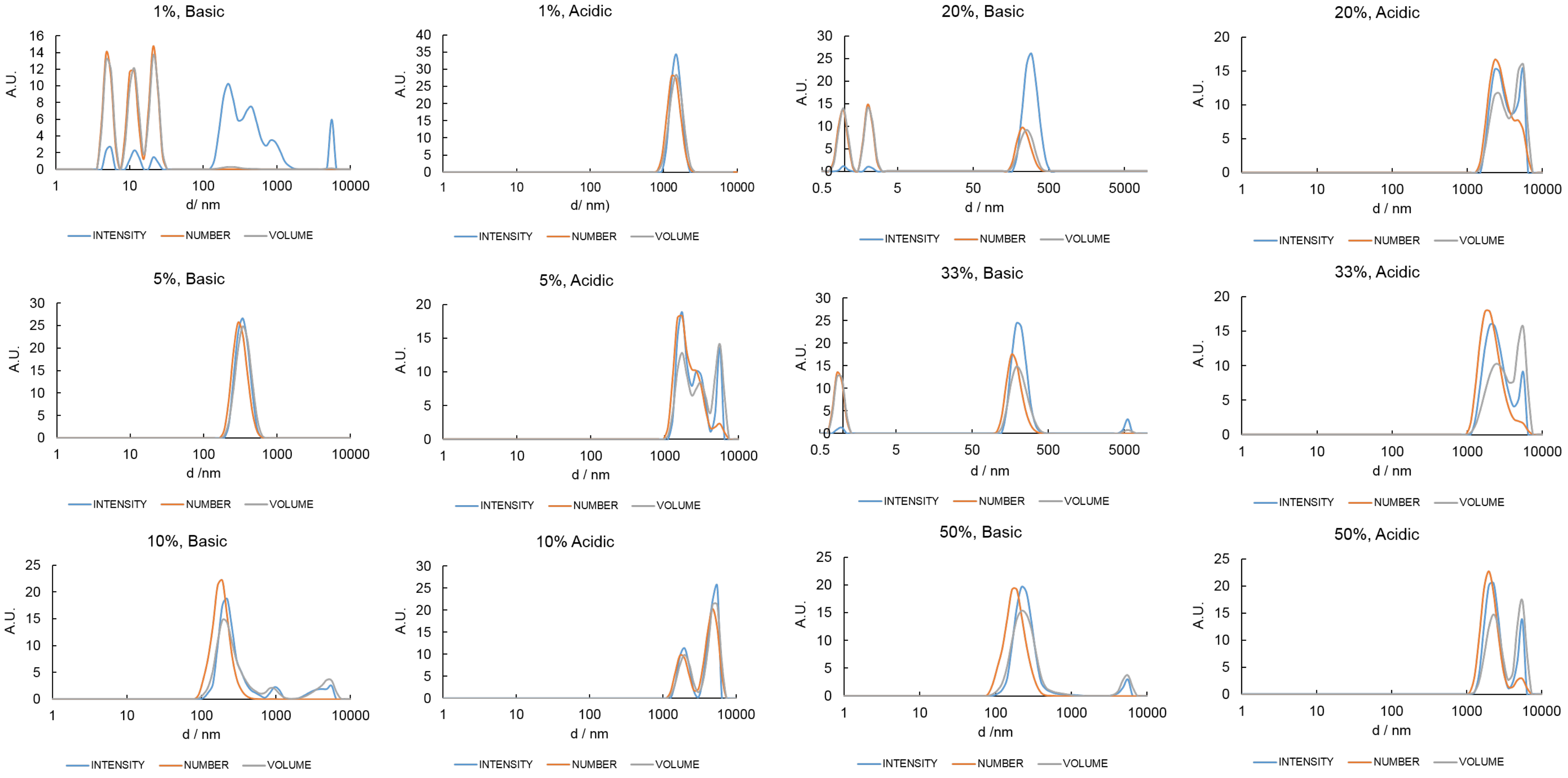

Fig. S9. Dynamic light scattering data for 50μM basic and acidic solutions of **OPV3-4T** coassemblies (*x* mol% **4T**).

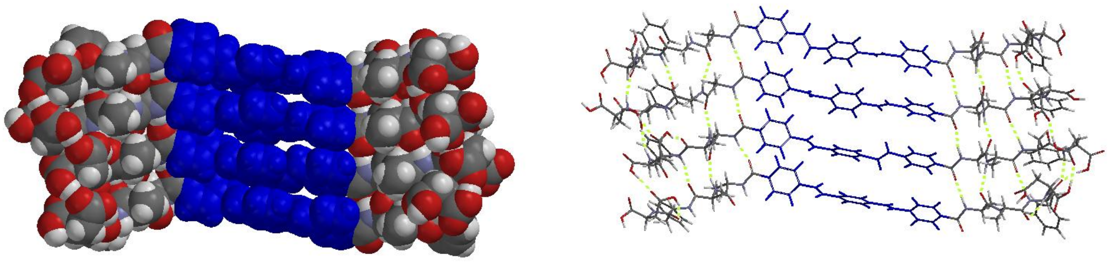

Fig. S10. Energy-minimized assembly model for a hypothetical portion of a HO-(DFAA)$_2$-**OPV3** assembly; (*left*) space-filling model and (*right*) wire structure showing H-bonds (---).

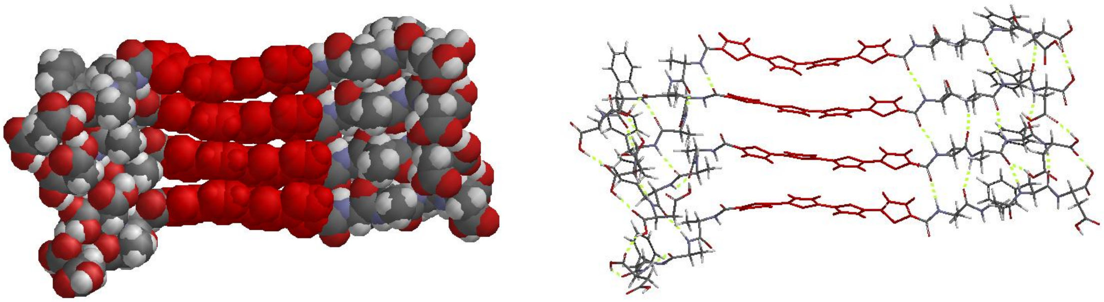

Fig. S11. Energy-minimized assembly model for a hypothetical portion of a HO-(DFAA)$_2$-**4T** assembly*;* (*left*) space-filling model and (*right*) wire structure showing H-bonds (---).

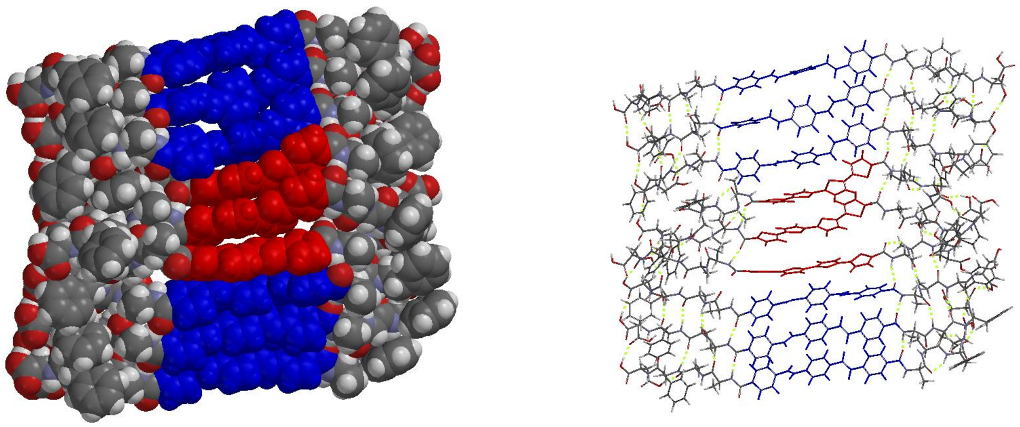

Fig. S12. Energy-minimized assembly model for a hypothetical portion of HO-(DFAA)$_2$-**4T** and –**OPV3** heterostructure with *multiple* **4T** units within the **OPV3** majority aggregate; (*left)* space-filling model and (*right*) wire structure showing H-bonds (---).

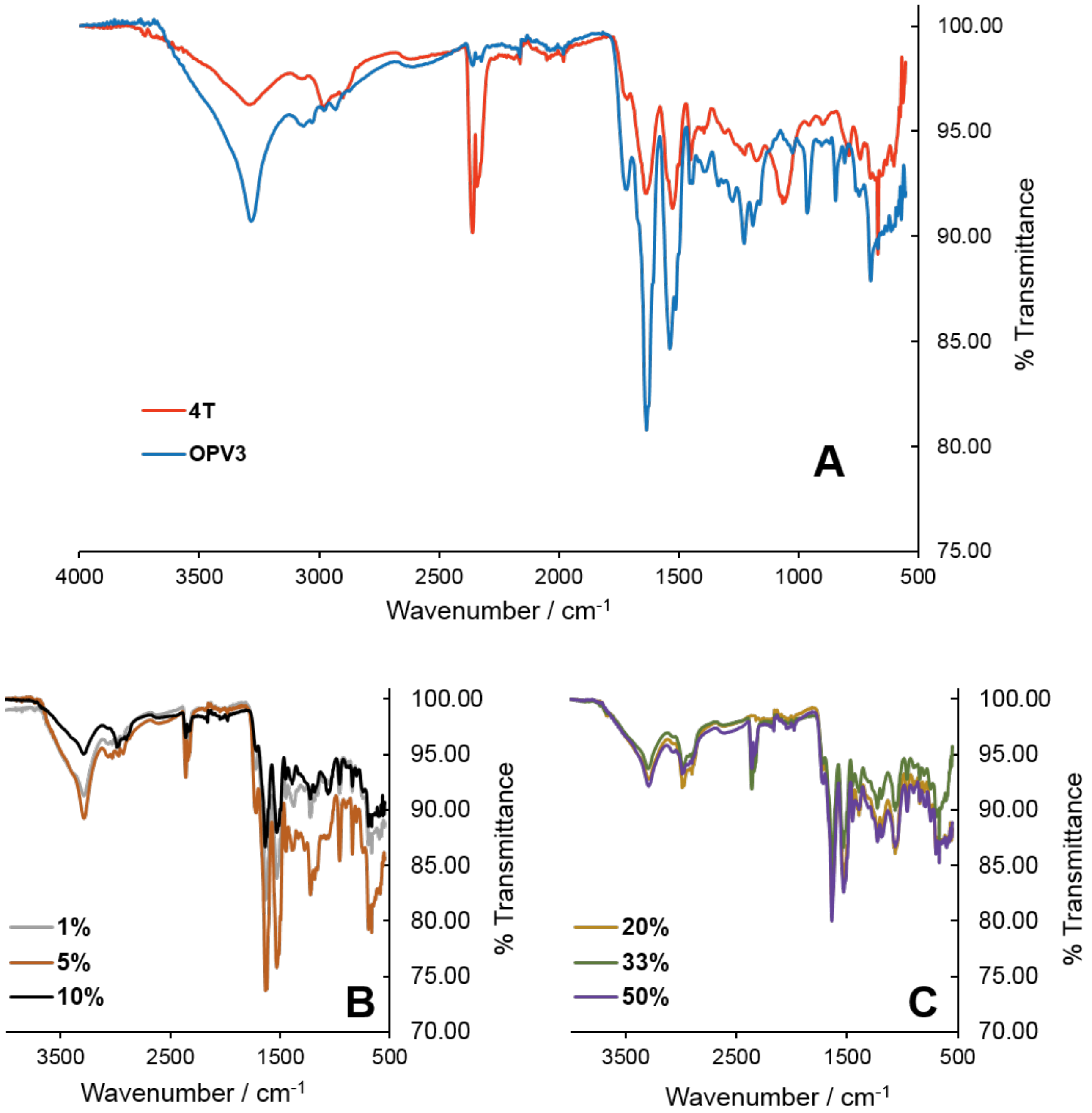

Fig. S13. ATR-IR spectra of lyophilized acidic solutions of (a) **OPV3**, **4T**, and (b,c) coassemblies.

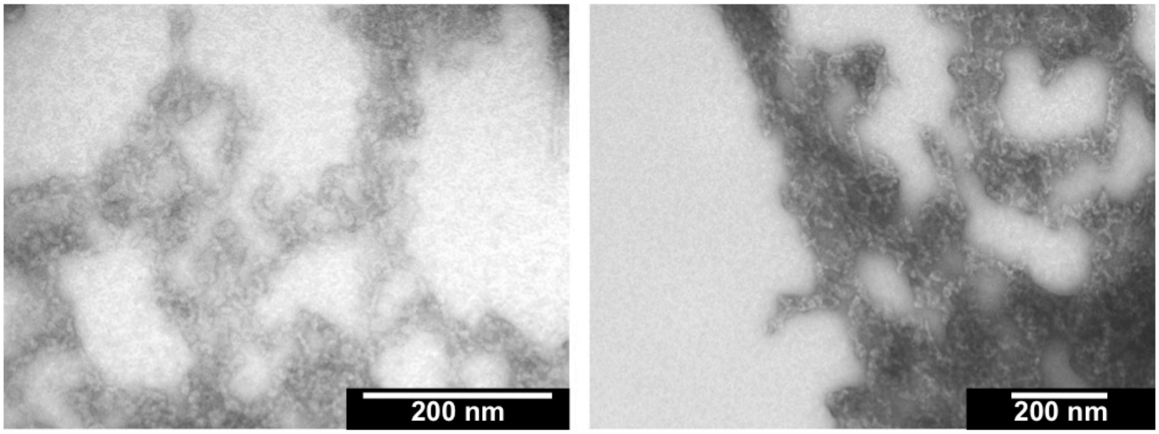

Fig. S14. Representative TEM images showing the nanostructures from a 0.1 wt% acidic solution of **4T.**

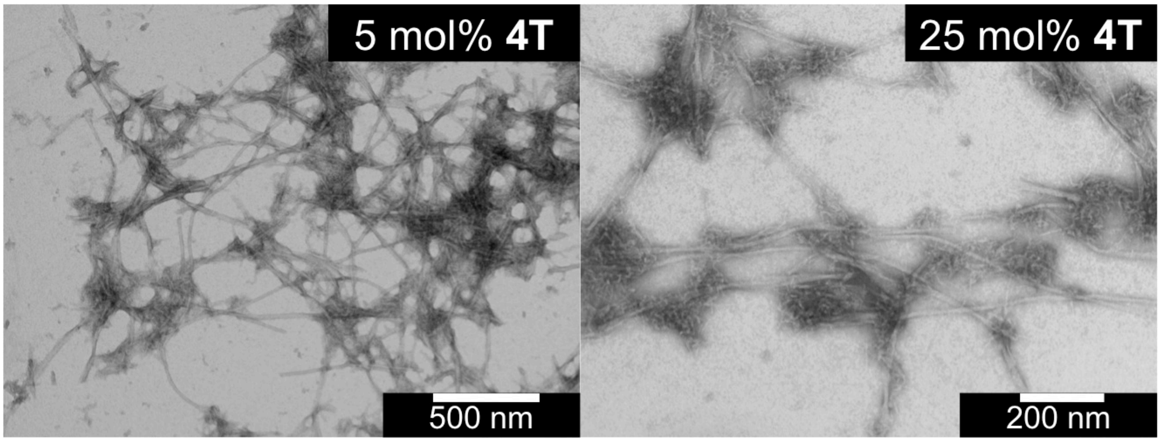

Fig. S15. Representative TEM images of the nanostructures from a 0.1 wt% acidic solution of coassembled **OPV3-4T** at different mol% **4T** (widths: 5 mol% **4T**= 10.8±1.6 nm; 25 mol% **4T**= 13.0±2.3 nm).

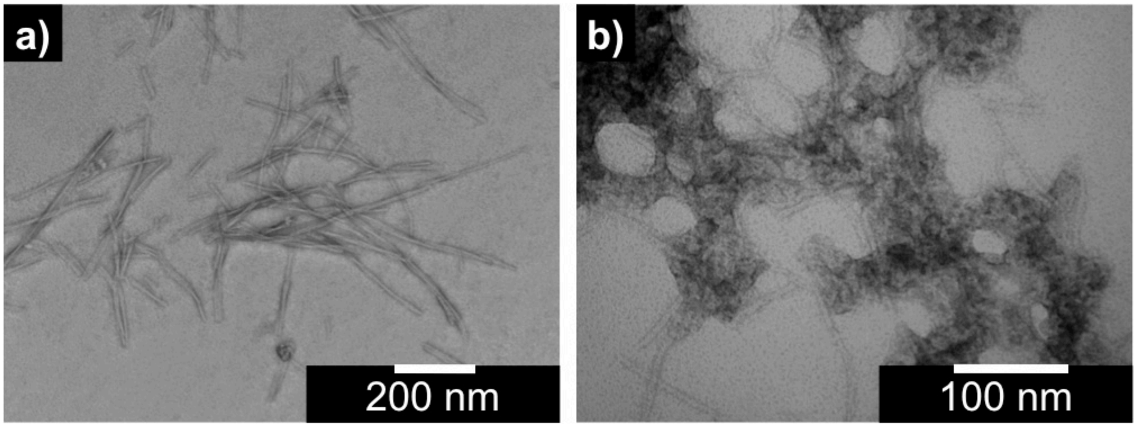

Fig. S16. Representative TEM images of the nanostructures from a 0.1 wt% acidic solution of (a) coassembled **OPV3-4T** (1:1) and a solution wherein (b) **4T** was added to pre-assembled **OPV3** solution (1:1).

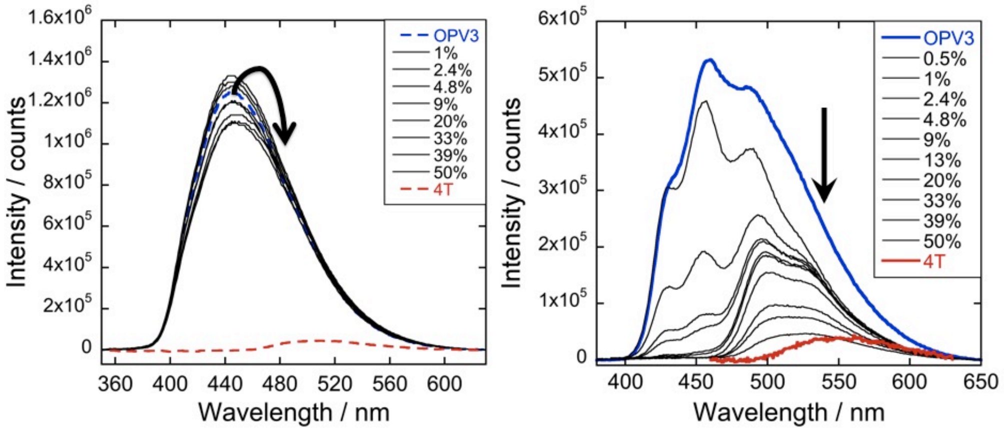

Fig. S17. Emission spectra (*ca.* pH 10, λ_{exc}=320 nm (*left*); *ca.* pH 2, λ_{exc}=330 nm, **4T** at 450 (*right*)) of **OPV3**, **4T**, and their coassemblies. These are the same solutions used in Fig. 3, showing all **OPV3:4T** coassembly ratios considered; arrow indicates the general trend as mol% **4T** increases.

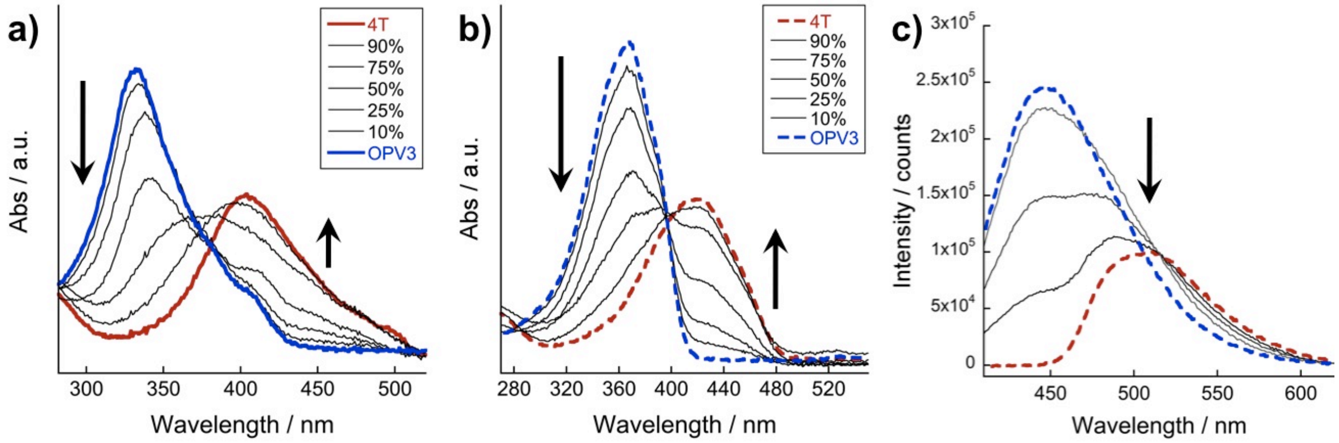

Fig. S18. UV-Vis (a,b) and PL (c) spectra for **OPV3:4T** mixtures, keeping the overall chromophore concentration constant under (a) acidic (—) and (b,c) basic (---) conditions; arrow indicates the general trend as mol% **4T** increases.

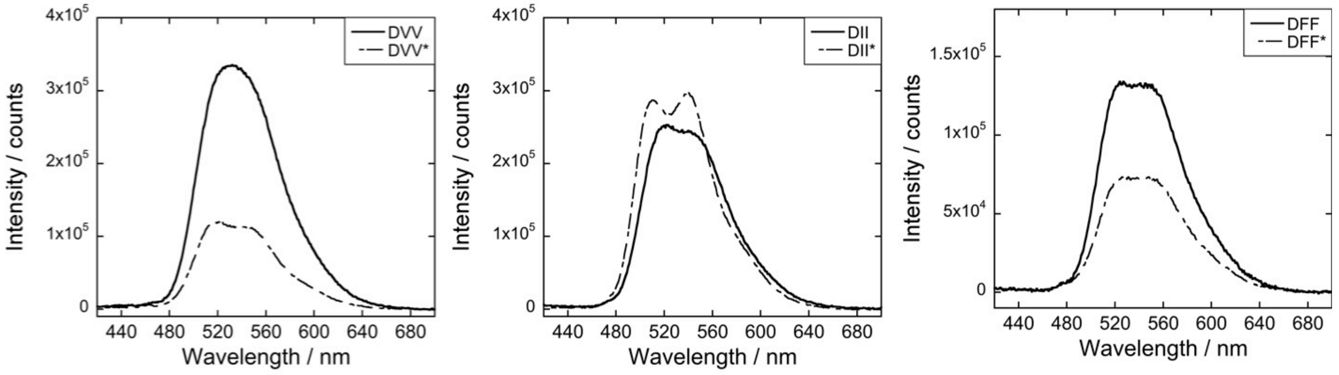

Fig. S19. Emission spectra of acidic and annealed HO-(D*XX*)$_2$-4T hydrophobic peptides (λ_{exc}=410 nm).

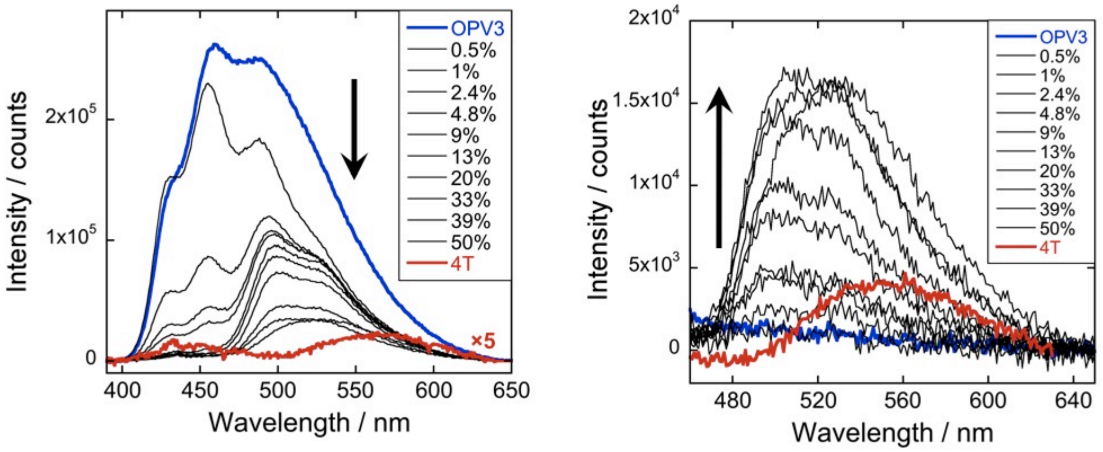

Fig. S20. Emission spectra of coassembled **OPV3-4T** acidic solutions, excited at 370 nm (*left*) and 450 nm (*right*); arrows indicate increasing mol% **4T**. These are the same acidic solutions used in Fig. 4, showing the absorption and emission spectra of all **OPV3:4T** coassembly ratios considered. (*Note that higher energy excitations such as 330 and 380 nm results to a higher energy spectral feature for pure, acidic, **4T** solution that does not correspond to the feature observed when excited near its λ_{max} (450 nm); hence, we used the feature observed at 450 nm excitation wavelength to assign 4T peaks in the spectra of the coassemblies.)*

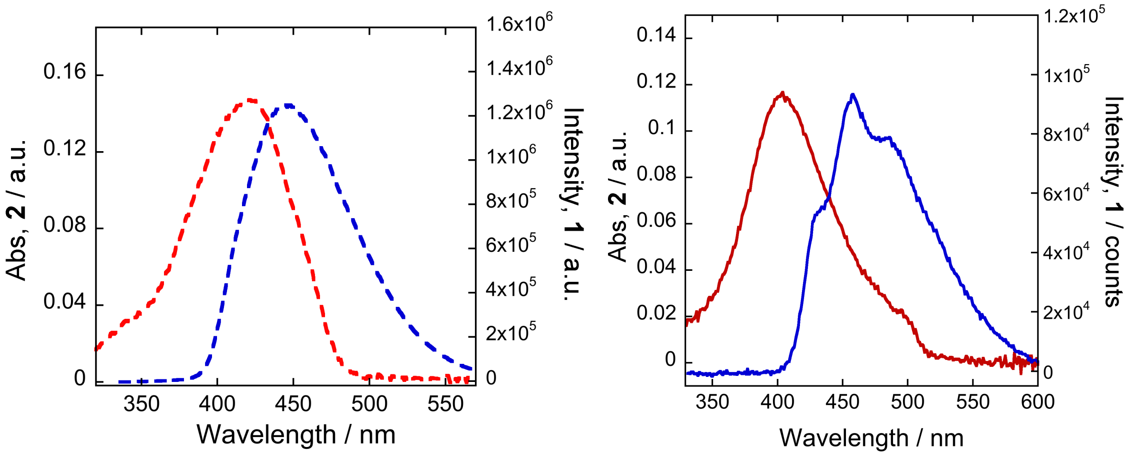

Fig. S21. Plots showing the spectral overlap between the donor emission and acceptor absorbance for basic (*left*) and (*right*) acidic solutions.

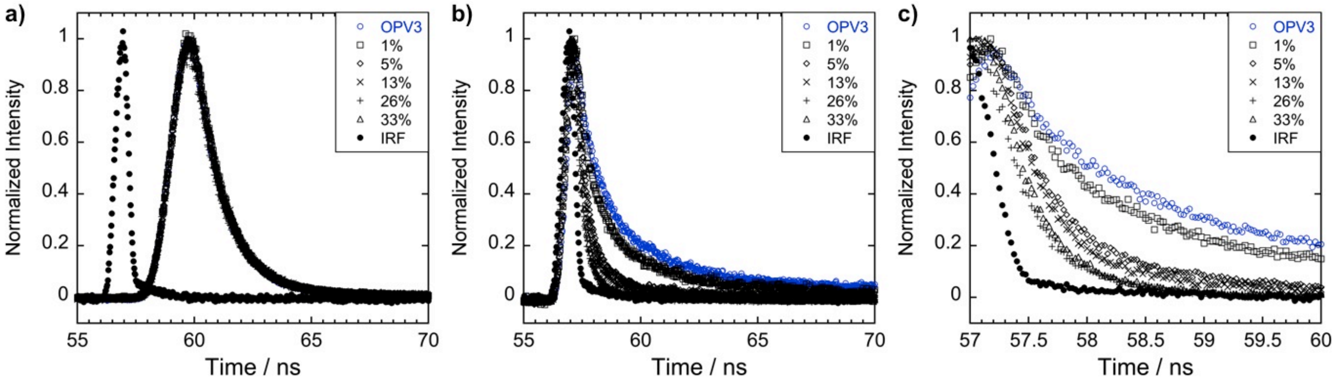

Fig. S22. Lifetime decay profiles (λ_{exc}= 375 nm) of **OPV3** and coassemblies under (a) basic (*ca.* pH 10) and (b,c) acidic (*ca.* pH 2) conditions.

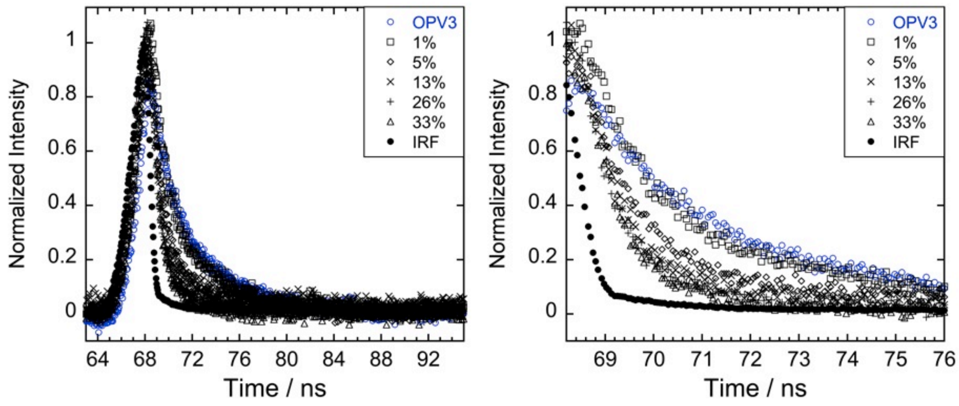

Fig. S23. Lifetime decay profiles (λ_{exc}= 340 nm) of **OPV3** and coassemblies under acidic (*ca.* pH 2) conditions.

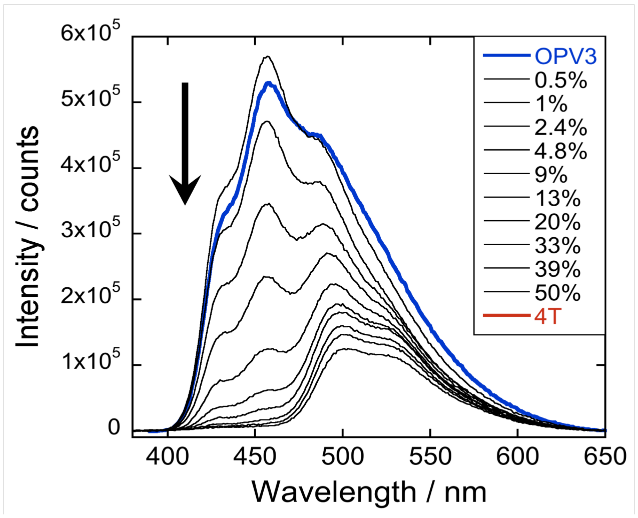

Fig. S24. Emission spectra (λ_{exc}=330 nm) of a solution wherein **4T** was titrated with acidic, pre-assembled **OPV3;** arrow indicates increasing mol% **4T**. These are the same acidic solutions used in Fig. 6, showing all **OPV3:4T** coassembly ratios considered.

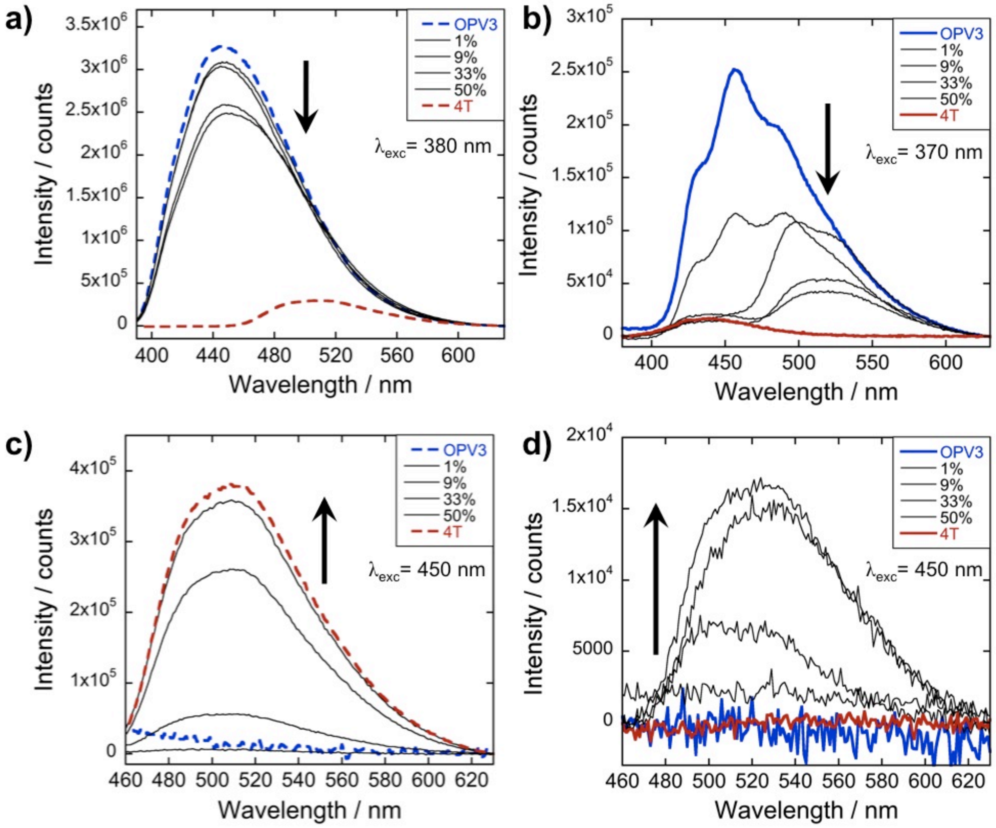

Fig. S25. *Reversibility of structure formation using pH control, different λ_{exc}*. Emission spectra when the re-basified (a,c) and reacidified (b,d) solutions are excited at different wavelengths. These are the same solutions used in Fig. 7, showing all **OPV3:4T** coassembly ratios considered; arrows indicate increasing mol% **4T**.

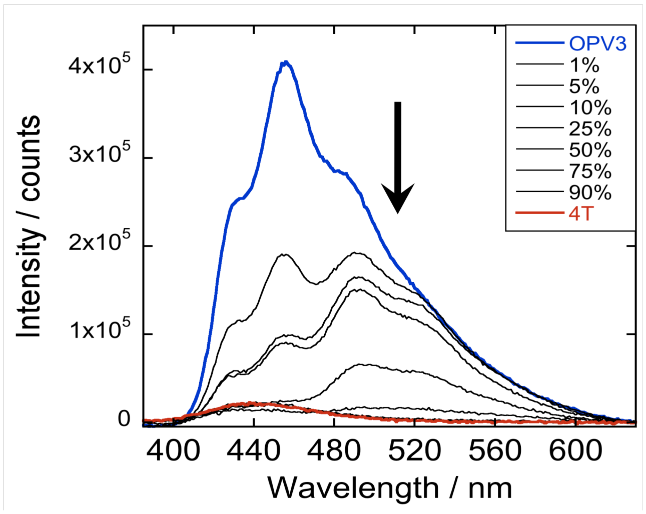

Fig. S26. *Titration with annealed samples.* PL spectra (λ_{exc}=375 nm) of annealed, acidic samples ([**OPV3**]= 7.4 μM); arrow indicates the trend as mol% **4T** increases.

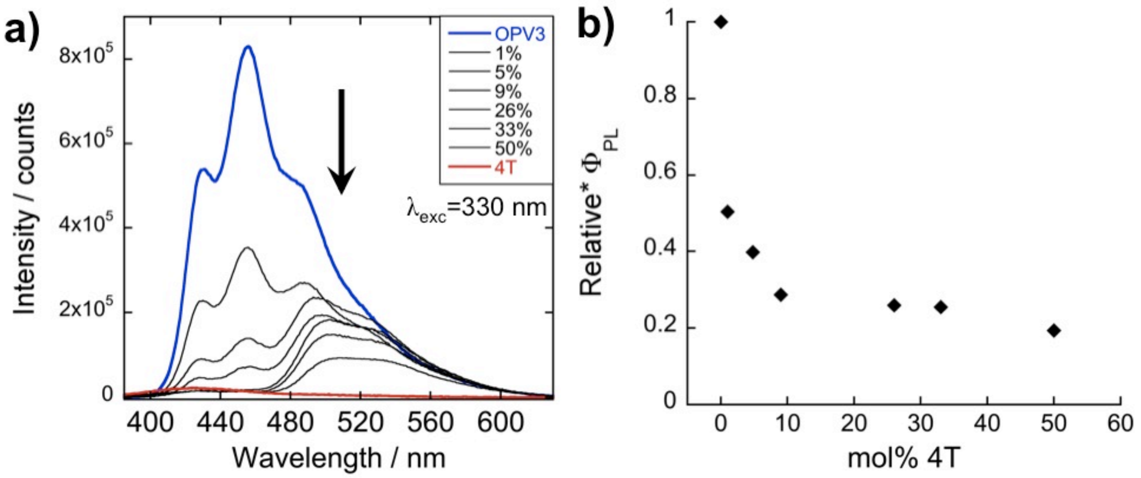

Fig. S27. (a) PL spectra (λ_{exc}=330 nm) of acidic samples ([**OPV3**]= 3.2 μM) and (b) the corresponding relative quantum yields* of **OPV3-4T** mixtures relative to **OPV3** after ~16 hours of sample preparation; arrow indicates the trend as mol% **4T** increases. At 1 mol% **4T**, the peak area decreased down to 54% with respect to acidic **OPV3.**

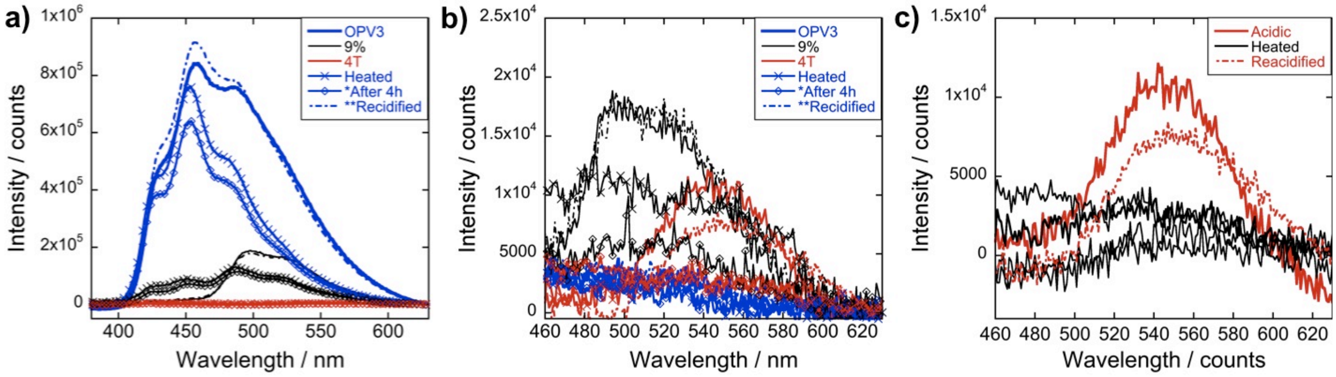

Fig. S28. PL monitoring of acidic **OPV3**, 9 mol % **4T** and **4T** solutions during the thermal cycling process (λ_{exc}= (a) 370 nm; (b) 450 nm); (c) showing **4T** emission when excited at 450 nm, including multiple time points during the 4h-cooling process.
